# Extraction, Structural Characterization and Biological Activities of Polysaccharides Derived From *Hericium erinaceus*

**DOI:** 10.1002/fsn3.71275

**Published:** 2025-11-25

**Authors:** Naxin Sun, Chao Li, Wu Liang, Zijian Wu, Xuemei Han, Suyun Xu

**Affiliations:** ^1^ Tianjin Key Laboratory of Food Biotechnology, College of Biotechnology and Food Science, Institute of Collaborative Innovation in Great Health Tianjin University of Commerce Tianjin China; ^2^ Tianjin Food Group Co., Ltd Tianjin China; ^3^ Tianjin Key Laboratory of Edible Probiotics, Tianjin InnoOrigin Biological Biotechnology Co., Ltd Tianjin China; ^4^ Key Laboratory of Agricultural Products Low Carbon Cold Chain, Ministry of Agriculture and Rural Affairs Tianjin China; ^5^ Department of Agriculture and Food Science Shijiazhuang University Shijiazhuang China

**Keywords:** β‐glucan, extraction, gut microbiota, *Hericium erinaceus*, lions mane mushroom, polysaccharides

## Abstract

*Hericium erinaceus* is acknowledged for its dual roles in medicinal and culinary contexts. The polysaccharides derived from *H. erinaceus* have attracted the attention of scientists owing to their bioactivities. This review focuses on the methodologies utilized for extraction and purification of *H. erinaceus* polysaccharides (HEP), as well as their structure and biological functions. We overview extraction and purification techniques, with hot water extraction and column chromatography being the most common. Subsequently, a thorough analysis of the structural characteristics of HEP was performed. The molecular weights of HEP ranged from 2.1 to 75,000 kDa. The monosaccharide composition of HEP was predominantly glucose. Additionally, the glycosidic bonds within HEP were primarily of the β‐glycosidic type, with β‐glucans characterized by 1 → 3 and 1 → 6 linkages representing the most prevalent structure. Furthermore, we summarize the biological activities and potential mechanistic pathways linked to HEP, which include immunomodulatory effects, antioxidant properties, hypoglycemic and hypolipidemic activities, as well as benefits in alleviating colitis and providing gastroprotective effects. Finally, we address the current challenges and limitations in the research surrounding *H. erinaceus* polysaccharides, while proposing promising directions for future investigations to further advance this area of study.

## Graphical abstract

This review summarizes the methods used to extract and purify *H. erinaceus* polysaccharides (HEP), primarily hot water extraction and column chromatography. Structural characterization indicates that the molecular weights of HEP range from 2.1 to 75,000 kDa, with glucose as the dominant monosaccharide and β‐glycosidic bonds, notably β‐glucans with 1 → 3 and 1 → 6 linkages. The main biological activities of HEP include immunomodulatory effects, antioxidant properties, hypoglycemic and hypolipidemic activities, as well as benefits in alleviating colitis and providing gastroprotective effects.

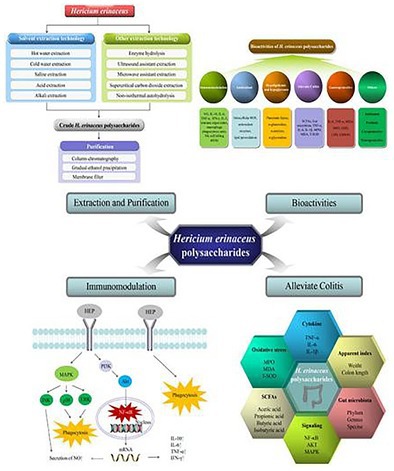

**Abbreviations**
EAE: enzyme assistant extraction of *H. erinaceus* polysaccharides
EHEP: enzymatic hydrolysis *H. erinaceus* polysaccharides
FHEP: fermented *H. erinaceus* polysaccharides
FJA, ZJA, and HBA: alkaline‐soluble polysaccharides obtained from fruit bodies of *H. erinaceus* from Fujian, Zhejiang, and Hubei provinces
FJW, ZJW, and HBW: water‐soluble polysaccharides obtained from fruit bodies of *H. erinaceus* from Fujian, Zhejiang, and Hubei provinces
GC–MS: gas chromatography–mass spectrometry
GEC: gel exclusion chromatography
HECP:
*H. erinaceus* crude polysaccharides
HEMP:
*H. erinaceus* polysaccharide extracted from mycelium
HEP:
*H. erinaceus* polysaccharides
HEP‐A:
*H. erinaceus* polysaccharides extracted by alkaline
HEP‐C:
*H. erinaceus* polysaccharides extracted by citric acid
HEP‐W:
*H. erinaceus* polysaccharides extracted by water
HPAEC: high‐performance anion exchange chromatography
HPLC: high‐performance liquid chromatography
HWE: hot water extraction of *H. erinaceus* polysaccharides
UPLC: ultra‐performance liquid chromatography

## 1 Introduction

Mushrooms have served as an important food source for millennia (Atmaca et al. 2024; Ferreira et al. 2010; Wang et al. 2019). The common edible mushrooms include *Hericium erinaceus*, *Pleurotus eryngii*, *Lentinus edodes*, *Agaricus bisporus*, *Flammulina velutipes*, *Ganoderma lucidum*, *Tremella fuciformis*, and so forth (Calabretti et al. 2021; El‐Ramady et al. 2022; Ezurike et al. 2023; Fu et al. 2025; Ganesan and Xu 2018; Park et al. 2023; Xie et al. 2025). The edible mushrooms are recognized not only for their palatability but also for their substantial nutritional composition, such as polysaccharides, proteins, dietary fibers, lipids, amino acids, water‐soluble vitamins, essential minerals, and so forth (Iqbal et al. 2024; Narmuratova et al. 2023; Paulauskiene et al. 2020; Wasser 2014; Xv et al. 2024). Furthermore, these edible mushrooms possess notable medicinal value, including antioxidant, antitumor, immunomodulatory, hypoglycemic, hypolipidemic and other bioactivities, which have increasingly attracted the attention of scientists.

**FIGURE 1:**
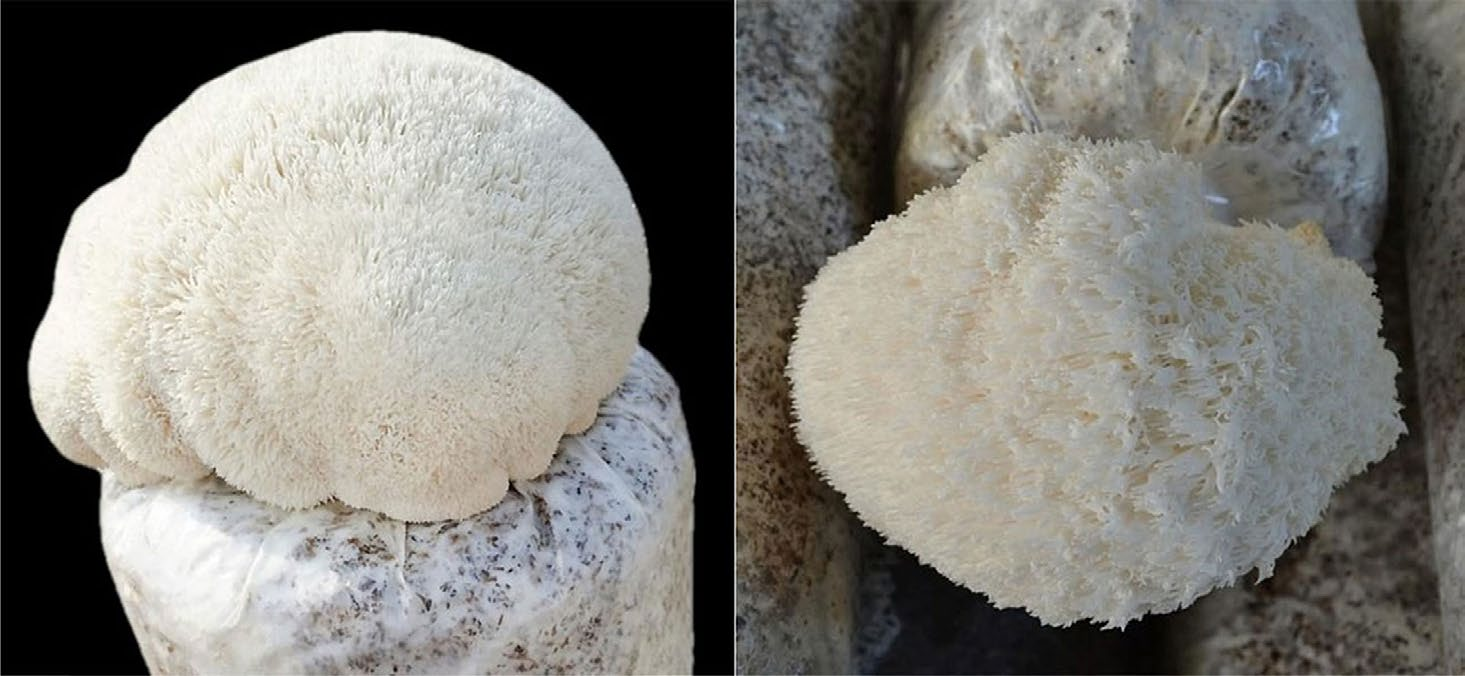
The fresh *H. erinaceus* samples (Yu et al. 2024).

*H. erinaceus* is a mushroom recognized for its dual utility in both medicinal applications and culinary practices (Yu et al. 2024), and it conforms to the theory of food and medicine homology, as it not only provides a feeling of fullness but also offers a range of biological benefits, including health maintenance, enhancement of well‐being, disease prevention, and potential therapeutic effects (Cong 2024). *H. erinaceus* exhibits a broad geographical distribution, predominantly found across Asia, North America and Europe, and it is also referred to as Houtougu (China), Yamabushitake (Japan), lion's mane, monkey head mushroom and white beard (He et al. 2017; Hoon et al. 2010; Lysakowska et al. 2025; Niu et al. 2024; Tan et al. 2024; Yanshree et al. 2022; Yuki et al. 2024). *H. erinaceus* serves as a significant reservoir of diverse biologically active constituents, encompassing polysaccharides, erinacines, hericenones, proteins, peptides, terpenes, ergosterol, and so forth. These active ingredients, especially polysaccharides, erinacines and hericenones, endow *H. erinaceus* with a range of bioactivities, such as antioxidant, anti‐aging, gastroprotective, liver‐protecting, immunomodulatory, neuroprotective, hypoglycemic, hypolipidemic, antitumor, sedative‐hypnotic and other beneficial activities (Gong et al. 2025; Gu et al. 2024; Jia et al. 2025; Lin et al. 2024; Liu et al. 2022; Qiu et al. 2024; Rui‐Qi et al. 2023; Wang et al. 2024) (Figure 1).

Polysaccharides are widely present in edible fungi, yeast, and so forth (Dong et al. 2024; Tingting et al. 2025). Prior research had demonstrated that the extraction efficiency of HEP could attain up to 25%, whereas the maximum polysaccharide extraction yields for *L. edodes*, *Phellinus linteus*, and *Morchella conica*, were reported as 14.11%, 19.49%, and 5.09%, respectively. These values were lower than the extraction rate observed for HEP (Chaiyasut et al. 2018; Sun et al. 2022). Furthermore, the molecular weight distribution of HEP exhibited considerable variability, spanning from 2.1 to 75,000 kDa, in comparison to other edible fungi polysaccharides, such as *L. edodes* (97.57 kDa—638.7 kDa), *P. linteus* (22 kDa—1700 kDa), *Suillellus luridus* (6.383 kDa—10.710 kDa) and *Oudemansiella radicata* (10.209 kDa—14.942 kDa) (Su et al. 2023; Sun et al. 2022; Wang et al. 2018). In addition to exhibiting a wide spectrum of molecular weights, HEP possessed distinctive molecular architectures relative to other polysaccharides. The predominant structure was β‐glucans characterized by 1 → 3 and 1 → 6 glycosidic linkages, which served as a fundamental basis for their bioactivities (Xie et al. 2021).

The fruiting body and mycelium represent distinct morphological forms of *H. erinaceus* . Research demonstrated that the total polysaccharide content in the fruiting body of *H. erinaceus* exceeded that found in the mycelium (He et al. 2017). Polysaccharides extracted from *H. erinaceus* have garnered significant interest owing to their bioactive properties. Research has demonstrated that *H. erinaceus* polysaccharides exhibit diverse bioactivities, such as gastric protection, alleviating colitis, immunomodulation, antioxidant, anti‐inflammatory activities and so on, and HEP are extensively utilized across various domains, including food, medicine, nutrition, cosmetics, and additional sectors (Ge et al. 2025). This review provides a comprehensive analysis of extraction protocols, purification techniques, structure, and bioactivities associated with *H. erinaceus* polysaccharides. The comprehensive analysis provides insights into the utilization of HEP in the development of pharmacological formulations and functional foods, thereby enhancing its industrial value chain.

## 2 Extraction and Purification of *Hericium Erinaceus* Polysaccharides

**FIGURE 2:**
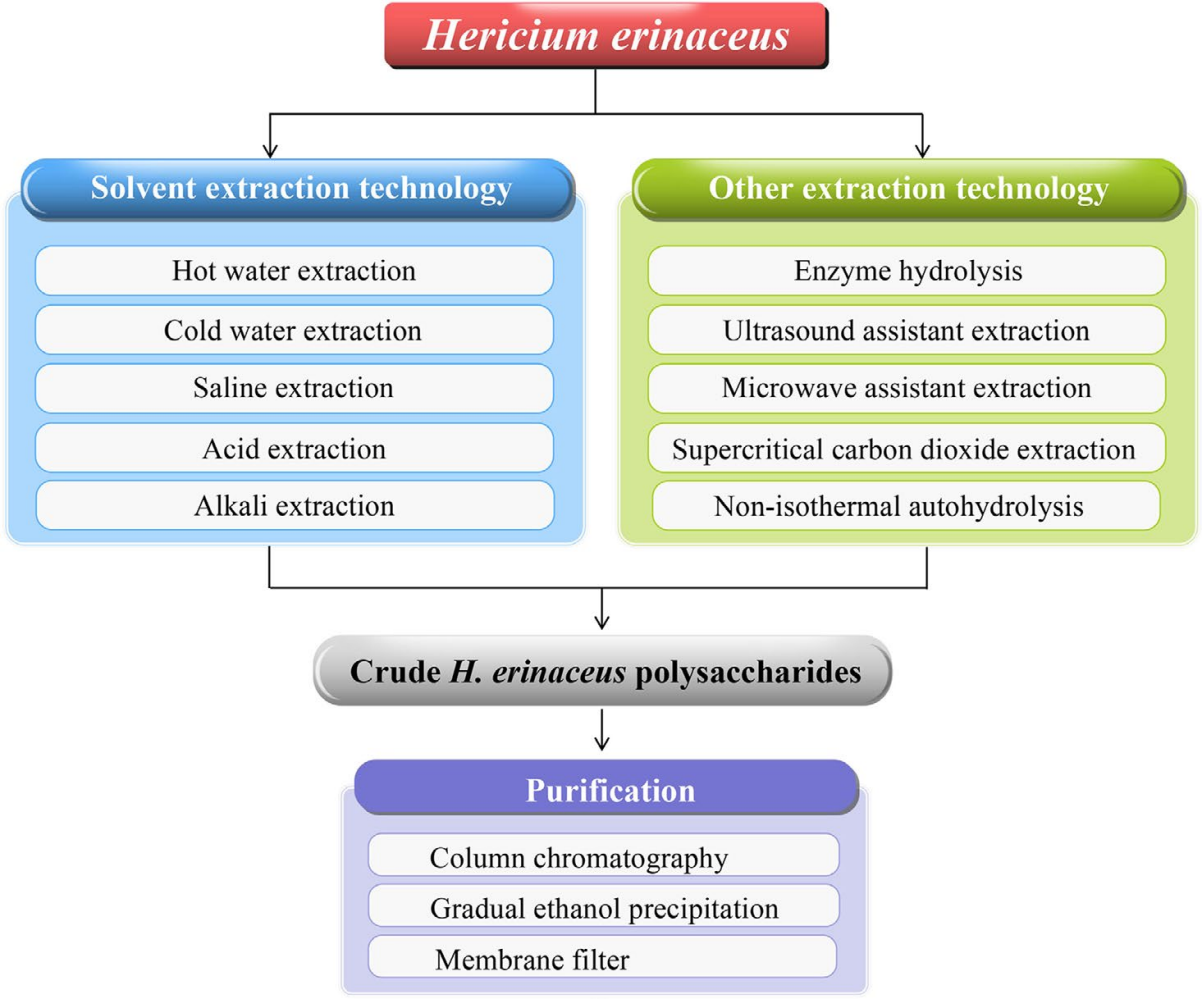
The extraction and purification of *H. erinaceus* polysaccharides.

There are several techniques for extracting *H. erinaceus* polysaccharides, such as hot water extraction (Chen et al. 2015; Cui et al. 2023; Han et al. 2013; He et al. 2022; Li et al. 2016; Liao et al. 2020; Qin et al. 2020; Ren, Sun, et al. 2023; Ren, Xu, et al. 2023; Shirokikh et al. 2020; Su et al. 2023; Tian et al. 2022; Wang et al. 2018, 2004; Wu et al. 2017, 2018; Xie et al. 2021; Yang et al. 2022), cold water extraction (Tu et al. 2021), saline extraction (Yan et al. 2018), acid extraction (Yan et al. 2018), alkali extraction (Chaiyasut et al. 2018; Chen et al. 2023; Zhuang et al. 2023), enzymatic hydrolysis (Deng et al. 2023; Liu et al. 2021; Qin et al. 2017; Zhu et al. 2014), ultrasound‐assisted enzymolysis (Yu et al. 2024), microwave‐assisted extraction, supercritical carbon dioxide extraction and non‐isothermal autohydrolysis (Parada et al. 2015). Subsequent chromatographic purification is essential to yield high‐purity *H. erinaceus* polysaccharides, predominantly achieved through column‐based separation techniques. In addition, gradual ethanol precipitation (Chen et al. 2023; Tian et al. 2022; Wang et al. 2018) and membrane filtration (He et al. 2022) are also used to purify *H. erinaceus* polysaccharides. Figure 2 and Table 1 systematically outline extraction methodologies and purification techniques of *H. erinaceus* polysaccharides.

**TABLE 1:** The methodologies employed for the extraction and purification of polysaccharides derived from *Hericium erinaceus*.

Name	Source	Extraction method	Extraction condition	Yield	Purification	Activities	References
HEP10	Fruiting bodies	Hot water	N/A	12.32%	Column chromatography: DEAE‐Sepharose Fast Flow, Sepharose G‐75	Immunomodulation; alleviate Colitis	(Ren, Sun, et al. 2023)
HEP‐30/50/70	Fruiting bodies	Hot water	Material: liquid = 1: 15, 95°C	2.03%, 1.87%; 1.69%	Gradual ethanol precipitation	Prebiotic	(Tian et al. 2022)
FHEP; HEP	Fruiting bodies	Hot water	Material: liquid = 1: 20, 85°C	3.7%; 2.4%	N/A	Antioxidant; hypoglycemic and hypolipidemic	(Su et al. 2023)
HEP	Fruiting bodies	Hot water	Material: liquid = 1: 20, 80°C	N/A	Membrane filter	N/A	(He et al. 2022)
HEMP	Mycelium	Hot water	Material: liquid = 1: 15, 80°C	N/A	N/A	Alleviate Colitis	(Ren, Xu, et al. 2023)
HEPs	Fruiting bodies	Hot water	75°C	2.735%	Column chromatography: DEAE‐52 cellulose, Sephadex G‐100	Immunomodulation	(Yang et al. 2022)
HEP	Fruiting bodies	Hot water	100°C	N/A	Column chromatography: DEAE‐52 cellulose, Sephadex G‐100	Antioxidant	(Qin et al. 2020)
HEP‐1	Fruiting bodies	Hot water	N/A	N/A	Column chromatography: DEAE‐52 cellulose, Sephadex G‐100	Hypoglycemic; hypolipidemic	(Cui et al. 2023)
HECP HERP	Fruiting bodies	Hot water	Material: liquid = 1: 15, 100°C	4.1%; 8.8%	Gradual ethanol precipitation	Alleviate Colitis	(Wang et al. 2018)
HP	Fruiting bodies	Hot water	100°C	8%	Column chromatography: DEAE‐Sepharose CL‐6B, Sephadex G‐100	N/A	(Wang et al. 2004)
HEP	Fruiting bodies	Hot water	Material: liquid = 1: 20, 85°C	2.56%	Column chromatography: DEAE‐Sepharose fast flow, Sephadex G‐100	Immunomodulation	(Wu et al. 2018)
HEP‐W	Fruiting bodies	Hot water	Material: liquid = 1: 20, 85°C	N/A	Column chromatography: DEAE‐Sepharose fast flow, Sephadex G‐100	Immunomodulation	(Wu et al. 2017)
HPB‐3	Fruiting bodies	Hot water	100°C	N/A	Column chromatography: Sephacryl S‐300	Immunomodulation	(Li et al. 2016)
HEP	Fruiting bodies	Hot water	N/A	N/A	N/A	Antioxidant	(Han et al. 2013)
HEP_N_	Fruiting bodies	Hot water	Material: liquid = 1: 15, 85°C	N/A	Column chromatography: DEAE‐52 cellulose	Antioxidant	(Liao et al. 2020)
PF	Fruiting bodies	Hot water	70°C	2.06%	N/A	Cryoprotective	(Shirokikh et al. 2020)
HEP3	Fruiting bodies	Hot water	Material: liquid = 1: 20, 100°C	5%	Column chromatography: DEAE‐Sepharose fast flow	N/A	(Xie et al. 2021)
HEP	Fruiting bodies	Hot water	90°C	N/A	N/A	Antioxidant; antitumor	(Chen et al. 2015)
HEP	Fruiting bodies	Cold water	Material: liquid = 1: 20, 4°C	4.29%	Column chromatography: Sephadex G‐150	Antioxidant	(Tu et al. 2021)
HEP‐W; HEP‐A; HEP‐C; HEP‐S	Fruiting bodies	Hot water; saline; acid; alkali	95°C; 95°C; 95°C; 25°C	8.10%; 11.76%; 8.84%; 9.66%	N/A	Antioxidant; hypoglycemic	(Yan et al. 2018)
HEP‐W; HEP‐A	Fruiting bodies	Hot water; alkali	Material: liquid = 1: 15, 100°C; material: liquid = 1: 10, 25°C	0.3%; 3.1%	N/A	Antioxidant; prebiotic	(Zhuang et al. 2023)
HEPs	Fruiting bodies	Hot water; alkali	Material: liquid = 1:15, 100°C; material: liquid = 1:10, 4°C	3.6%–6%; 1%–3.2%	Gradual ethanol precipitation	N/A	(Chen et al. 2023)
BG	Fruiting bodies	Alkali	80°C	25%	N/A	Immunomodulation	(Chaiyasut et al. 2018)
EHEP	Fruiting bodies	Enzymatic hydrolysis	50°C	N/A	N/A	Immunomodulation	(Liu et al. 2021)
HEPs	Fruiting bodies	Hot water; enzymatic hydrolysis	100°C; 40°C	3.6%–4%	N/A	N/A	(Deng et al. 2023)
HEP	Mycelium	Enzymatic hydrolysis	N/A	13.9%	Column chromatography: DEAE‐52 cellulose	Antitumor	(Qin et al. 2017)
EAE; HWE	Fruiting bodies	Enzymatic hydrolysis; hot water	Material: liquid = 1: 30	13.46%; 8.03%	N/A	N/A	(Zhu et al. 2014)
SDF	Fruiting bodies	Ultrasound assistant enzymolysis	27°C	12.48%	N/A	Hypolipidemic	(Yu et al. 2024)
HEP	Fruiting bodies	Microwave assistant extraction; supercritical carbon dioxide extraction; enzyme hydrolysis; non‐isothermal autohydrolysis	N/A	N/A	N/A	N/A	(Parada et al. 2015)

Abbreviation: N/A: not available.

### 2.1 Solvent Extraction Technology

Hot water extraction represents the most prevalent and widely adopted methodology for polysaccharide isolation in contemporary research. Ren, Sun, et al. (2023) successfully isolated crude polysaccharides from *H. erinaceus* (designated as HECP) through the hot water extraction method. Then HECP was subsequently solubilized in distilled water and subjected to deproteinization through the Savage method. The obtained polysaccharides underwent purification through sequential chromatography using DEAE‐Sepharose Fast Flow (1.6 × 10 cm) and Sepharose G‐75 (1 × 30 cm) columns, with elution performed using distilled water and NaCl solution, respectively. The eluted substance was subjected to dialysis and freeze‐dry procedures in preparation for subsequent analysis. The purified compound was named HEP10 with a calculated yield of 12.32% and a total sugar content of 89.03% relative to the starting material. Tian et al. (2022) successfully isolated three polysaccharides from *H. erinaceus* . The fruiting bodies of *H. erinaceus* were ground into powders, which were then treated twice with distilled water (1:15, *w*/*v*) at 95°C for 3 h. Then the resulting extracts were centrifuged, concentrated, deproteinized and further purified by the method of gradual ethanol precipitation. Adjusting the proportion of 95% ethanol to obtain different ethanol concentrations (70%, 50%, and 30%) for precipitating crude *H. erinaceus* polysaccharides. The resulting polysaccharides were designated as HEP‐70, HEP‐50, and HEP‐30, with yields of 1.69%, 1.87%, and 2.03%, and purities of 95.18%, 71.8%, and 54.36%, respectively. Su et al. (2023) inoculated *Lactobacillus gasseri* JM1 into *H. erinaceus* solution, and then the fermented solution was lyophilized into powder. The powder was used to extract polysaccharides at 85°C for 3.5 h. After precipitating with 95% ethanol, the precipitate underwent centrifugation to isolate crude polysaccharides. Then the crude polysaccharides underwent deproteinization utilizing the trichloroacetic acid (TCA) method, followed by decolorization with a 3% hydrogen peroxide solution. Subsequently, the samples underwent dialysis in distilled water and were freeze‐dried to achieve a powdered form. The extraction rates of FHEP and HEP were 3.7% and 2.4%, respectively. It was worth mentioning that the polysaccharide contents of FHEP and HEP were both more than 93%. He et al. (2022) mixed the *H. erinaceus* powder with water, and the proportion of raw material to water was established at 1:20. The resultant mixture was heated at 80°C for a duration of 4 h to facilitate the extraction of polysaccharides from *H. erinaceus* . Then they purified HEP by ordinary membrane filter and collected the freeze‐dried samples. In addition to fruiting bodies, polysaccharides could be extracted from the mycelium of this species using a hot water extraction method. The mycelium of *H. erinaceus* underwent hydrothermal treatment twice at 80°C for 2 h. The resulting extract was subsequently gathered and concentrated. The concentrate was immersed in 80% ethanol overnight to precipitate *H. erinaceus* mycelium polysaccharides, resulting in a total sugar content of 42% (Ren, Xu, et al. 2023).

Beyond the predominant technique of hot water extraction, alternative solvents were employed to extract polysaccharides. Tu et al. (2021) extracted *H. erinaceus* polysaccharides using a cold water extraction method. *H. erinaceus* was washed, dried, smashed and sieved to produce a uniform powder. The powder underwent a dual extraction process utilizing deionized water for 6 h at 4°C. Subsequently, 95% ethanol was introduced to facilitate the precipitation of polysaccharides. Following this, centrifugation was performed, and deproteinization was carried out using Sevage reagent, resulting in the collection of crude polysaccharides. Then the obtained crude polysaccharides were dissolved in water and purified by Sephadex G‐150 (1.6 × 40 cm) column. The crude HEP yield was measured at 4.29%, while the purified HEP had a total sugar content of 96.43%. Yan et al. (2018) employed various solutions to isolate polysaccharides from *H. erinaceus* , utilizing hot water (95°C), NaOH/NaBH4 (25°C), citric acid (95°C, pH 3.0) and NaCl (95°C). The polysaccharides obtained through these processes were designated as HEP‐W (water), HEP‐A (alkaline), HEP‐C (citric acid), and HEP‐S (sodium chloride). Their respective yields were 8.10%, 11.76%, 8.84%, and 9.66%. The total sugar contents for each were 72.3%, 79.17%, 81.51%, and 73.97%, respectively. Zhuang et al. (2023) successfully isolated two distinct polysaccharides from *H. erinaceus* : a water‐soluble polysaccharide designated as HEP‐W and an alkali‐soluble polysaccharide referred to as HEP‐A. The extraction of HEP‐W was conducted using distilled water (1:15, g/mL) at 100°C for 2 h. In contrast, HEP‐A was extracted utilizing a NaOH/NaBH4 solution (1:10, g/mL) at 25°C for 3 h. The yields obtained for HEP‐W and HEP‐A were measured at 0.3% and 3.1%, respectively, with corresponding total sugar contents of 76.69% and 83.92%. Chen et al. (2023) extracted *H. erinaceus* polysaccharides using hot water and an alkali solution. The fruit bodies powder of *H. erinaceus* from Fujian, Zhejiang, and Hubei underwent two rounds of boiling water extraction followed by centrifugation. The water extraction method obtained three water‐soluble polysaccharides, designated as FJW, ZJW, and HBW. Then the water‐insoluble residue was subsequently extracted by the NaOH/NaBH_4_ solution, resulting in the isolation of three alkali‐soluble polysaccharides, referred to as FJA, ZJA, and HBA. The water‐soluble polysaccharides were sequentially purified through gradual ethanol precipitation using different ethanol concentrations. Chaiyasut et al. (2018) extracted beta‐glucan (BG) by the alkali extraction method. The *H. erinaceus* was immersed in NaOH solution and incubated at 80°C. After shaking and centrifugation, the obtained pellets were subsequently suspended in acetic acid, continuously stirred at 80°C for 2 h, and centrifuged at 4°C. Then the obtained samples were dried using anhydrous ethanol and a hot air oven. The extraction rate and beta‐glucan content were 25% and 87%, respectively.

### 2.2 Other Extraction Technology

The method of enzymatic hydrolysis is frequently employed for polysaccharide extraction. Liu et al. (2021) extracted polysaccharides from *H. erinaceus* through enzymatic hydrolysis, utilizing *endo*‐rhamnosidase. In their procedure, the polysaccharide extract was combined with 20.3 U of *endo*‐rhamnosidase and subjected to digestion at a temperature of 50°C for 1 h. Subsequently, the resulting mixtures were heated to 100°C for 10 min to inactivate the enzyme. After dialysis, concentration, and ethanol precipitation, the precipitate was freeze‐dried and collected for further experiments. Deng et al. (2023) first extracted *H. erinaceus* polysaccharides by the method of hot water extraction, and then they hydrolyzed HEP with 1,3‐β‐glucanase. The HEP and 1,3‐β‐glucanase (2.0 U/mL) were incubated at 40°C for different times. After incubation, the enzyme was inactivated by heating (80°C, 20 min). Qin et al. (2017) employed an enzyme‐assisted extraction technique to isolate polysaccharides from *H. erinaceus* . The powder was combined with a solution of compound enzymes, specifically cellulase, pectinase and papain (2:1:1), and subsequently subjected to digestion under specified conditions. Through the analysis of single‐factor and response surface experiments, they identified optimal extraction parameters: extraction temperature 50°C, duration time 79 min, pH 5.7, and water‐to‐raw material ratio 33.4 mL/g. Under the above optimal parameters, the extraction yield of *H. erinaceus* polysaccharides was determined to be 13.9%. Following extraction, the HEP solution underwent further purification using a DEAE‐52 cellulose column (2.6 × 40 cm), resulting in the separation of three distinct polysaccharides. Notably, HEP‐2 emerged as the predominant fraction, constituting 66.5% of the total yield. Zhu et al. (2014) employed an enzyme‐assisted extraction technique to isolate polysaccharides from *H. erinaceus* , while the types and proportions of enzymes (cellulose: pectinase: trypsin = 2:2:1) differed from Qin et al. (2017). The optimal parameters for extraction were: extraction temperature 52.03°C, extraction duration 33.79 min, and pH 5.71. The yield and polysaccharide content of EAE under optimal extraction parameters were 13.46% and 44.19%, respectively.

In addition to the methods of solvent extraction and enzymic hydrolysis extraction, scientists have also used ultrasound‐assisted enzymolysis (Yu et al. 2024), microwave‐assisted extraction, supercritical carbon dioxide extraction, and non‐isothermal autohydrolysis to obtain polysaccharides from *H. erinaceus* (Parada et al. 2015). Soluble dietary fiber (SDF) is a significant bioactive polysaccharide found in *H. erinaceus* . Yu et al. (2024) isolated SDF using ultrasound‐assisted enzymatic extraction (UAEE). Mushroom powder was mixed with water and heated in a water bath, followed by centrifugation. The resulting filtrate was then hydrolyzed using Lywallzyme. The study identified the optimal extraction parameters, which included: Lywallzyme concentration 1.0%, ultrasonication duration 35 min, ultrasonication power 150 watts, and complex protease concentration 1.2%. Under these conditions, the yield and purity of soluble dietary fiber were 12.48% and 89.04%, respectively. Parada et al. (2015) employed a series of stages utilizing environmentally friendly solvents to obtain extracts containing polysaccharide and phenolic fractions from *H. erinaceus* . The sequential extraction process comprised microwave‐assisted extraction conducted at 400 W, supercritical carbon dioxide extraction performed at 20 MPa pressure and 40°C temperature with the addition of 10% ethanol, enzymatic hydrolysis extraction utilizing protease, cellulase, and pectinase at 50°C for a duration of 3 h, and non‐isothermal autohydrolysis extraction carried out at 200°C.

In conclusion, various techniques exist for the extraction of *H. erinaceus* polysaccharides, such as solvent (hot water, cold water, saline, acid, alkali) extraction, enzymatic hydrolysis, ultrasound or microwave‐assisted extraction, supercritical carbon dioxide extraction, and non‐isothermal autohydrolysis. The extraction yields of different methods were as follows: 0.3% to 12.32% for hot water, 4.29% for cold water, 11.76% for saline, 8.84% for acid, 1% to 25% for alkali, 3.6% to 13.9% for enzymatic hydrolysis, and 12.48% for ultrasound‐assisted enzymolysis. Among these techniques, hot water extraction is the most frequently utilized technique. The hot water extraction approach is favored in polysaccharides preparation, not owing to its technological advancement, but because it provides an optimal balance between cost‐effectiveness and safety. This technique necessitates minimal equipment investment and is easy to scale up. Furthermore, it employs hot water exclusively as the solvent, resulting in no residual contaminants, thereby meeting the stringent safety requirements mandated in the food and pharmaceutical industries. However, it is also associated with certain drawbacks, such as elevated temperatures, prolonged processing times, and low extraction efficiency (Sun et al. 2023). To address these limitations, several novel extraction methods are applied to extract polysaccharides. The enzymatic hydrolysis extraction technique operates under mild conditions, while extraction techniques utilizing ultrasound or microwave‐assisted extraction, as well as supercritical carbon dioxide extraction are characterized by their rapid processing times and high extraction efficiencies (Nai et al. 2021). These innovative techniques facilitate the straightforward, environmentally friendly, efficient, and cost‐effective separation of polysaccharides, thereby establishing a foundation for the subsequent purification processes of *H. erinaceus* polysaccharides.

## 3 Structural Characteristics of *Hericium erinaceus* Polysaccharides

Polysaccharides are macromolecular compounds composed of many monosaccharide molecules connected through glycosidic bonds. The physical–chemical properties of polysaccharides are influenced by several factors, including their molecular weights, monosaccharide compositions, chemical structures, and conformational features (Ji, Hou, et al. 2020; Ji, Yin, et al. 2020). The molecular weights can be determined using high‐performance gel permeation chromatography (HPGPC) and high‐performance liquid chromatography (HPLC), while the monosaccharide compositions can be analyzed through ion chromatography (IC), gas chromatography (GC), and high‐performance anion exchange chromatography (HPAEC), etc. The conformational features of polysaccharides can be assessed via scanning electron microscopy (SEM) and atomic force microscopy (AFM), and the chemical structures can be elucidated using nuclear magnetic resonance (NMR), and so forth. Table 2 presents the structure of polysaccharides derived from *H. erinaceus* .

**TABLE 2:** The structure of polysaccharides derived from *Hericium erinaceus*.

Name	Mw	Monosaccharide compositions	Structural characteristics	Instrument	References
HEP10	9.9 kDa	Glu: Gal: Ara: Fuc: Man: Xyl = 84.36%: 7.11%: 5.72%: 0.85%: 0.91%: 1.05%	Might encompass both α‐glycosidic and β‐glycosidic linkages, specifically involving (1 → 2) and (1 → 6) glycosidic connections	HPGPC; HPAEC; NMR	(Ren, Sun, et al. 2023)
FHEP; HEP	35 kDa; 75,000 kDa	Fuc: Ara: Gal: Glu: Man: GluUA = 3.96%: 0.14%: 12.54%: 80.68%: 1.33%: 1.35%; Fuc: Ara: Gal: Glu: Xyl: Man: Rib: GluUA: ManUA = 7.90%: 0.28%: 24.18%: 56.74%: 2.30%: 5.87%: 1.00%: 1.27%: 0.46%	Mainly α‐type linkage; FHEP exhibited an increased presence of side‐chain polysaccharides characterized by a greater degree of branching; HEP had no branched chains	GEC; NMR; SEM	(Su et al. 2023)
HEP‐1/2/3/4/5	2120 kDa; 927 kDa; 110 kDa; 19.9 kDa; 10.9 kDa	Fuc: Glu: Gal = 7.87: 23.72: 68.41	HEP‐1 consisted of (1 → 6)‐linked‐galactose; HEP‐2/3/4/5 were characterized by (1 → 3)‐linked glucosyl residues or (1 → 6)‐linked α‐D‐galactosyl residues	UPLC; GC–MS; NMR	(Yang et al. 2022)
HEP	43 kDa	Primarily composed of rhamnose and glucose	The principal chain was characterized by the structure (→6)‐β‐D‐Glcp‐(1→), which was linked to a branched structure consisting of (→2)‐α‐L‐Rhap‐(1→)	SEM; AFM; GC–MS; LC; NMR	(Qin et al. 2020)
HEP‐1	16.7 kDa	Rha: GluUA: Glu: Gal: Fuc = 0.41: 0.017: 1.82: 67.14: 1: 0.28	Comprised of (→3,6)‐β‐D‐Glcp‐(1→), β‐D‐Glcp‐(1→), (→3)‐β‐D‐Glcp‐(1→) and (→6)‐β‐D‐Glcp‐(1→)	GPC; HPLC; NMR	(Cui et al. 2023)
HPA; HPB	50 kDa; 30 kDa	Fuc: Gal: Glc = 0.423: 2.110: 1; Glc: Gal = 11.529: 1	The backbone of HPA consisted of (1 → 6)‐linked galactose, while HPB was hypothesized to consist of (1 → 6)‐linked glucose; β configuration	GC; GC–MS	(Wang et al. 2004)
HEP‐S	18.3 kDa	Fuc: Man: Glu: Gal: Rha = 0.93: 1.36: 8.68: 4.08: 1.47	(1 → 3,6)‐α‐L‐Rhamnose, (1 → 3,4)‐α‐D‐Glucose, (1 → 3,4)‐β‐D‐Mannose, (1 → 6)‐α‐D‐Galactose, (1 → 2)‐β‐L‐Fucose and (1→)‐α‐D‐Glucose	HPGPC; GC; NMR	(Wu et al. 2018)
HEP‐W	15.9 kDa	Gal: Glu: Man: Fuc: Rha = 7.06: 5.60: 0.89: 1.59: 0.98	T‐β‐Gal, (1 → 3,6)‐α‐D‐Glc, (1 → 3,4)‐β‐D‐Man, (1 → 2,6)‐α‐D‐Gal, (1 → 3)‐α‐Rha, (1 → 2)‐β‐L‐Fuc and (1→)‐α‐D‐Glc	HPGPC; GC; NMR	(Wu et al. 2017)
HPB‐3	15 kDa	Fuc: Gal: Glu = 5.2: 23.9: 1.1	Consisted of α‐(1 → 6)‐linked galactopyranosyl units	HPLC; HPAEC; GC–MS; NMR	(Li et al. 2016)
HEP_N_	12.713 kDa	Man: Glu: Gal = 5.13%: 43.02%: 51.85%	(1 → 3,6)‐linked mannose, (1 → 6)‐linked galactose, (1 → 6)‐linked mannose, (1 → 6)‐linked glucose, (1 → 4)‐linked glucose and (1→)‐linked glucose; both α‐type and β‐type linkages	HPGPC; SEM; GC; NMR	(Liao et al. 2020)
HEP3	13.3 kDa	Only composed of glucose	Characterized by a structure comprising multiple glucan chains linked primarily through 1 → 6 glycosidic bonds, with interconnections facilitated by one or two 1 → 3 linked glucose units; α configuration	NMR; HPAEC	(Xie et al. 2021)
HEP	19.7 kDa	GlcA: Man: Glu: Gal: Fuc = 0.01: 0.12: 0.09: 2.87: 1	α‐1‐6‐Gal and α‐1‐4‐Fuc linkages, which were further branched with (→1,6)‐Glc and (→3,6)‐α‐D‐Man‐(1→) units	HPGPC; IC; NMR; SEM	(Tu et al. 2021)
HEPs	5–30 kDa	Primarily consisted of glucose	The primary chain of heterogalactans was composed of α‐(1 → 6)‐Galp residues, whereas the predominant structure of heteroglucans consisted of β‐(1 → 3)‐Glcp, β‐(1 → 4)‐Glcp and β‐(1 → 6)‐Glcp residues	HPGPC; SEM; GC–MS; NMR	(Chen et al. 2023)
EHEP	5.6 kDa	Glu: Rha: Gal: Man = 53.5%: 10.0%: 0.4%: 1.2%	The main chain was (→6) β‐D‐Glcp‐(1→) branched with α‐L‐Rhap‐(1→) and β‐D‐Glcp‐(1→)	SEM; AFM; GC–MS; NMR	(Liu et al. 2021)
HECP HERP; RP‐10/50/60/70/80/S	362.7 and 18.8 kDa; 15.9 kDa; N/A; 20.7 kDa; 17.4 kDa; 9.0 kDa; 4.4 kDa; 2.1 kDa	HECP, HERP: Glc, Gal, Fuc; RP‐10/70: Gal, Glc; RP‐50/60: Fuc, Gal, Glc; RP‐80/S: Glc	The anomeric carbon could exhibit both α‐type and β‐type linkages	HPAEC; HPLC; SEM	(Wang et al. 2018)
HEP‐W/S/C/A	597.6 and 314.7 kDa; 449.4 and 442.7 kDa; 229.8 and 263.6 kDa; 256.8 and 320.8 kDa	Composed of Rha, Ara, Man, Gal and Glu	Mainly β‐glycosidic bonds	GC； SEM	(Yan et al. 2018)
HEP‐W; HEP‐A	Approximately 60 kDa	Mainly composed of glucose	Pyran ring polysaccharide containing β‐glycosidic linkage	HPGFC； IC	(Zhuang et al. 2023)
HEMP	4.6 kDa	Man: Glu: Gal = 6.5%: 32.38%: 52.56%	N/A	HPGPC; HPLC	(Ren, Xu, et al. 2023)
HEP‐2	38.76 kDa	Consisted of Glu, Gal, Man, Fuc, Ara, Xyl and Rha	N/A	HPAEC; GPC	(Qin et al. 2017)
HEP	N/A	Xyl: Rib: Glu: Ara: Gal: Man = 7.8%: 2.7%: 68.4%: 11.3%: 2.5%: 5.2%	N/A	HPLC	(Han et al. 2013)
PF	N/A	Rha: Fru: Xyl: Ara: Man: Glu: Gal = 7: 8: 1: 19: 14: 26: 27	N/A	GC	(Shirokikh et al. 2020)
EAE; HWE	N/A	Man: Glu: Xyl: Gal = 15.16: 5.55: 4.21: 1 Man: Glu: Xyl: Gal = 11.99: 6.48: 2.6: 1	Pyranose form containing α‐type linkage	SEM; AFM; GC	(Zhu et al. 2014)

Abbreviation: N/A: not available.

In recent years, researchers have conducted analyses on the molecular weights, monosaccharide compositions, and structure of polysaccharides derived from *H. erinaceus* (refer to Table 2). Ren, Sun, et al. (2023) obtained the *H. erinaceus* polysaccharides HEP10. The monosaccharide compositions of HEP10 were glucose (Glu), fucose (Fuc), arabinose (Ara), galactose (Gal), mannose (Man) and xylose (Xyl) with respective percentages of 84.36%, 7.11%, 5.72%, 0.85%, 0.91% and 1.05%. The HMR indicated that HEP10 might encompass both α‐glycosidic and β‐glycosidic linkages, specifically involving (1 → 2) and (1 → 6) glycosidic connections. Su et al. (2023) fermented *H. erinaceus* polysaccharides by *Lactobacillus gasseri* JM1 and obtained degraded *H. erinaceus* polysaccharides (FHEP). The molecular weights (Mw) of FHEP and HEP were 35 and 75,000 kDa, respectively. FHEP primarily consisted of the following monosaccharides: Fuc, Ara, Gal, Glu, Man and glucuronic acid (GluUA) with respective percentages of 3.96%, 0.14%, 12.54%, 80.68%, 1.33% and 1.35%. While the monosaccharide compositions of HEP were Gal, Ara, Fuc, Glu, Man, Xyl, Rib, GluUA and ManUA with percentages of 24.18%, 0.28%, 7.90%, 56.74%, 5.87%, 2.30%, 1.00%, 1.27% and 0.46%. The results demonstrated that the fermentation process led to a reduction of both the molecular weights and the polysaccharide compositions of *H. erinaceus* polysaccharides. Additionally, it was observed that the FHEP exhibited an increased presence of side‐chain polysaccharides characterized by a greater degree of branching. Yang et al. (2022) successfully extracted five distinct *H. erinaceus* polysaccharides. The molecular weights of them were 2120 kDa (HEP‐1), 927 kDa (HEP‐2), 110 kDa (HEP‐3), 19.9 kDa (HEP‐4) and 10.9 kDa (HEP‐5), respectively. The monosaccharide compositions of the HEPs were found to include fucose, glucose and galactose (molar ratio = 7.87: 23.72: 68.41). The structural analysis revealed that the backbone of HEP‐1 was primarily composed of (1 → 6)‐linked galactose with branching occurring at the *O*‐2 position. In contrast, HEP‐2/3/4/5 were characterized by (1 → 3)‐linked glucosyl residues with branching at the *O*‐6 position, or (1 → 6)‐linked α‐D‐galactosyl residues, which also exhibited branches at the *O*‐2 position. The polysaccharides derived from *H. erinaceus* , as extracted by Qin et al. (2020), were primarily comprised of glucose and rhamnose. The principal chain of HEP was characterized by the structure (→6)‐β‐D‐Glcp‐(1→), which was associated with a branched structure consisting of (→2)‐α‐L‐Rhap‐(1→). The polysaccharide HEP‐1 extracted by Cui et al. (2023) was composed of GluUA, Gal, Glu, Fuc and Rha, and the molar ratio was 0.017: 67.14: 1.82: 1: 0.28: 0.41. The molecular weight of HEP‐1 was 16.7 kDa, and its composition was as follows: (→3,6)‐β‐D‐Glcp‐(1→), β‐D‐Glcp‐(1→, →3)‐β‐D‐Glcp‐(1→) and (→6)‐β‐D‐Glcp‐(1→). Wang et al. (2004) isolated two water‐soluble *H. erinaceus* polysaccharides, designated as HPA and HPB. The molecular weight of HPA was 50 kDa, and that of HPB was 30 kDa. HPA was primarily composed of fucose, galactose and glucose, and the molar ratio was 0.423: 2.110: 1. While HPB was determined to consist of the monosaccharides glucose and galactose, and the molar ratio was 11.529: 1. The backbone of HPA consisted of (1 → 6)‐linked galactose units with branching at the *O*‐2 position of certain galactose residues. In contrast, the backbone of HPB was hypothesized to consist of (1 → 6)‐linked glucose with branching occurring at the O‐3 position. Wu et al. (2018) obtained a polysaccharide HEP‐S with a molecular weight of 18.3 kDa. The monosaccharide composition of HEP‐S was Fuc, Man, Glu, Gal and Rha, and the molar ratio was 0.93: 1.36: 8.68: 4.08: 1.47. The predominant types of linkages present in HEP‐S were identified as (1 → 3,6)‐α‐L‐Rhamnose, (1 → 3,4)‐α‐D‐Glucose, (1 → 3,4)‐β‐D‐Mannose, (1 → 6)‐α‐D‐Galactose, (1 → 2)‐β‐L‐Fucose and (1→)‐α‐D‐Glucose. A novel heteropolysaccharide, designated as HEP‐W, was extracted by Wu et al. (2017). It was comprised of Gal, Glu, Man, Fuc and Rha, and the molar ratio was 7.06: 5.60: 0.89: 1.59: 0.98. The primary types of glycosidic linkages present in HEP‐W included T‐β‐Gal, (1 → 3,6)‐α‐D‐Glc, (1 → 3,4)‐β‐D‐Man, (1 → 2,6)‐α‐D‐Gal, (1 → 3)‐α‐Rha, (1 → 2)‐β‐L‐Fuc and (1→)‐α‐D‐Glc. The heteropolysaccharide HPB‐3 extracted by Li et al. (2016) was characterized by a composition of fucose, galactose and glucose in a molar ratio of 5.2: 23.9: 1.1 with a molecular weight of 15 kDa. The structural backbone of HPB‐3 consisted of α‐(1 → 6)‐linked galactopyranosyl units, which featured a side chain of α‐L‐fucopyranose attached at the *O*‐2 position. Liao et al. (2020) successfully isolated a novel polysaccharide designated as HEP_N_, and the molecular weight of HEP_N_ was 12.713 kDa. Monosaccharide compositional results indicated that HEP_N_ was comprised of Man, Glu and Gal in the following proportions: 5.13%, 43.02% and 51.85%, respectively. Furthermore, HEP_N_ exhibited six distinct linkage types, specifically: (1 → 3,6)‐linked mannose, (1 → 6)‐linked galactose, (1 → 6)‐linked mannose, (1 → 6)‐linked glucose, (1 → 4)‐linked glucose and (1→)‐linked glucose. Xie et al. (2021) successfully isolated a water‐soluble β‐glucan designated as HEP3, and the molecular weight of HEP3 was 13.3 kDa. The composition of HEP3 was exclusively glucose, characterized by a structure comprising multiple glucan chains linked primarily through 1 → 6 glycosidic bonds, with interconnections facilitated by one or two 1 → 3 linked glucose units. Tu et al. (2021) obtained a novel *H. erinaceus* polysaccharide, and the molecular weight of HEP was 19.7 kDa. Fucose, galactose, glucose, mannose and gluconic acid were found to be the major monosaccharides in HEP, and their molar ratio was 1: 2.87: 0.09: 0.12: 0.01. HEP was characterized as a pyranose with an α‐configuration. Its primary structure consisted of α‐1‐6‐Gal and α‐1‐4‐Fuc linkages, which were further branched with (→1,6)‐Glc and (→3,6)‐α‐D‐Man‐(1→) units. Chen et al. (2023) isolated polysaccharides from *H. erinaceus* sourced from various regions, specifically Zhejiang, Hubei and Fujian. The molecular weights of these polysaccharides varied between 5 and 30 kDa, and they were primarily composed of glucose. In addition, HEPs demonstrated various structural configurations characterized by distinct glycosidic linkage types, including glucan, fucoglucan, glucofucogalactan and fucogalactoglucan. The primary chain of heterogalactans was composed of α‐(1 → 6)‐Galp residues, whereas the predominant structure of heteroglucans consisted of β‐(1 → 3)‐Glcp, β‐(1 → 4)‐Glcp and β‐(1 → 6)‐Glcp residues. The enzymatic hydrolysis polysaccharides (EHEP) extracted by Liu et al. (2021) revealed a composition comprising glucose, rhamnose, galactose and mannose with respective proportions of 53.5%, 10.0%, 0.4% and 1.2%. The Mw of EHEP was 5.6 kDa, and the main chain was (→6) β‐D‐Glcp‐(1→) branched with α‐L‐Rhap‐(1→) and β‐D‐Glcp‐(1→).

Owing to the complexity of *H. erinaceus* polysaccharides' structure, some research on HEP only stayed at the stage of simple structure exploration. Wang et al. (2018) obtained eight *H. erinaceus* polysaccharides. The *H. erinaceus* crude polysaccharides (HECP) were subjected to a comprehensive purification protocol that included deproteinization, dialysis, and freeze‐drying, resulting in the production of *H. erinaceus* refined polysaccharides (HERP). Subsequently, the HERP underwent a systematic series of fractional separations utilizing a gradual ethanol precipitation method. Adjusting the proportion of ethanol to obtain different ethanol concentrations (10%, 50%, 60%, 70%, and 80%) for precipitating HERP. The resulting polysaccharides were designated as RP‐10/50/60/70/80. The supernatant resulting from the last procedure was labeled as RP‐S. Molecular weight measurement indicated that HECP exhibited two primary peaks, with the molecular weights of peak 1 and peak 2 measured at 362.7 and 18.8 kDa, respectively. Other polysaccharides had only one peak and the Mw of HERP, RP‐S, RP‐80/70/60/50 were 15.9, 2.1, 4.4, 9.0, 17.4 and 20.7 kDa, respectively. Monosaccharide compositional results indicated that HECP and HERP were primarily comprised of glucose, galactose and fucose. In RP‐10 and RP‐70, galactose and glucose were identified as the predominant monosaccharides. Conversely, RP‐50 and RP‐60 exhibited fucose, galactose and glucose as the major monosaccharides. Furthermore, RP‐80 and RP‐S were predominantly composed of glucose. Yan et al. (2018) extracted four water‐soluble *H. erinaceus* polysaccharides. Molecular weight measurement indicated that all HEPs had two main peaks, and the molecular weights were 597.6 and 314.7 kDa (HEP‐W), 449.4 and 442.7 kDa (HEP‐S), 229.8 and 263.6 kDa (HEP‐C), 256.8 and 320.8 kDa (HEP‐A). In addition, HEP‐W/S/C/A were comprised of Rha, Ara, Man, Gal and Glu, and the molar ratio was 9.9: 1.6: 1.0: 6.1: 35.5, 1.7: 1.0: 1.0: 4.6: 30.7, 9.0: 2.0: 1.0: 7.5: 40.7 and 3.3: 2.4: 1.0: 9.5: 66.5, respectively, and the preliminary structural experiments showed that the main bond type of HEPs was β‐glycosidic bonds. Zhuang et al. (2023) employed two distinct methodologies to extract polysaccharides: water extraction method, resulting in water‐extracted *H. erinaceus* polysaccharides (HEP‐W), and alkali extraction method, yielding alkali‐extracted *H. erinaceus* polysaccharides (HEP‐A). The monosaccharide compositions of HEP‐W were Fuc, glucosamine, Gal, Glu, Man and GluUA. While HEP‐A was comprised of Ara, Rha, Fuc, glucosamine, Glu, Gal and GluUA. Both polysaccharides were classified as heteropolysaccharides, with glucose as their predominant component. Furthermore, they were characterized as pyranose ring polysaccharides that feature β‐glycosidic linkages. Ren, Xu, et al. (2023) obtained *H. erinaceus* mycelium‐derived polysaccharide (HEMP) with a molecular weight of 4.6 kDa. The monosaccharide composition of HEMP consisted of galactose, glucose and mannose with respective percentages of 52.56%, 32.38% and 6.5%. The *H. erinaceus* mycelia polysaccharides (HEP‐2) extracted by Qin et al. (2017) consisted of Glu, Gal, Man, Fuc, Ara, Xyl and Rha with a molar ratio of 42.05: 21.31: 13.07: 12.47: 0.96: 8.76:1.38, and the molecular weight of HEP‐2 was 38.76 kDa. The HEP extracted by Han et al. (2013) consisted of Xyl, Rib, Glu, Ara, Gal and Man with percentages of 7.8%, 2.7%, 68.4%, 11.3%, 2.5% and 5.2%. Shirokikh et al. (2020) extracted polysaccharide from *H. erinaceus* BP 16. The predominant monosaccharides identified in the PF were Rha, Fru, Xyl, Ara, Man, Glu and Gal, and the molar ratio was 7: 8: 1: 19: 14: 26: 27, respectively. Zhu et al. (2014) employed two distinct methodologies to extract polysaccharides from *H. erinaceus* : hot water extraction and enzyme‐assisted extraction. The monosaccharide compositions of EAE and HWE were Glu, Xyl, Gal and Man, and the molar ratio was 5.55: 4.21: 1: 15.16 and 6.48: 2.6: 1: 11.99, and they were pyranose form containing α‐type linkage.

The structure–activity relationship (SAR) of polysaccharides is essential to elucidate the correspondence between the chemical structure of polysaccharides and their specific bioactivities. The primary structure of HEP constitutes the fundamental structural element influencing its structure–activity relationship and serves as the foundation for elucidating its biological functions. This primary structure encompasses characteristics such as molecular weight and its distribution, monosaccharide composition, types of glycosidic linkages, as well as the position and length of branching chains (Sun et al. 2024). Research indicates that high molecular weight HEP typically exhibits immunological activity, whereas HEP with lower molecular weights tends to demonstrate enhanced antioxidant properties (Zhang et al. 2024). Furthermore, the composition and proportion of various monosaccharides can influence the activities of polysaccharides; for instance, HEP containing glucose as a constituent tends to exhibit enhanced biological functions. Additionally, the types of glycosidic linkages and the presence of branched chains influence the activity of HEP by modulating its spatial conformation and structural stability. The sophisticated architecture of HEP plays a crucial role in modulating its functional expression, with the triple helix conformation potentially being associated with its immunomodulation and antitumor properties (Wang et al. 2019).

## 4 Bioactivities of *Hericium erinaceus* Polysaccharides

**FIGURE 3:**
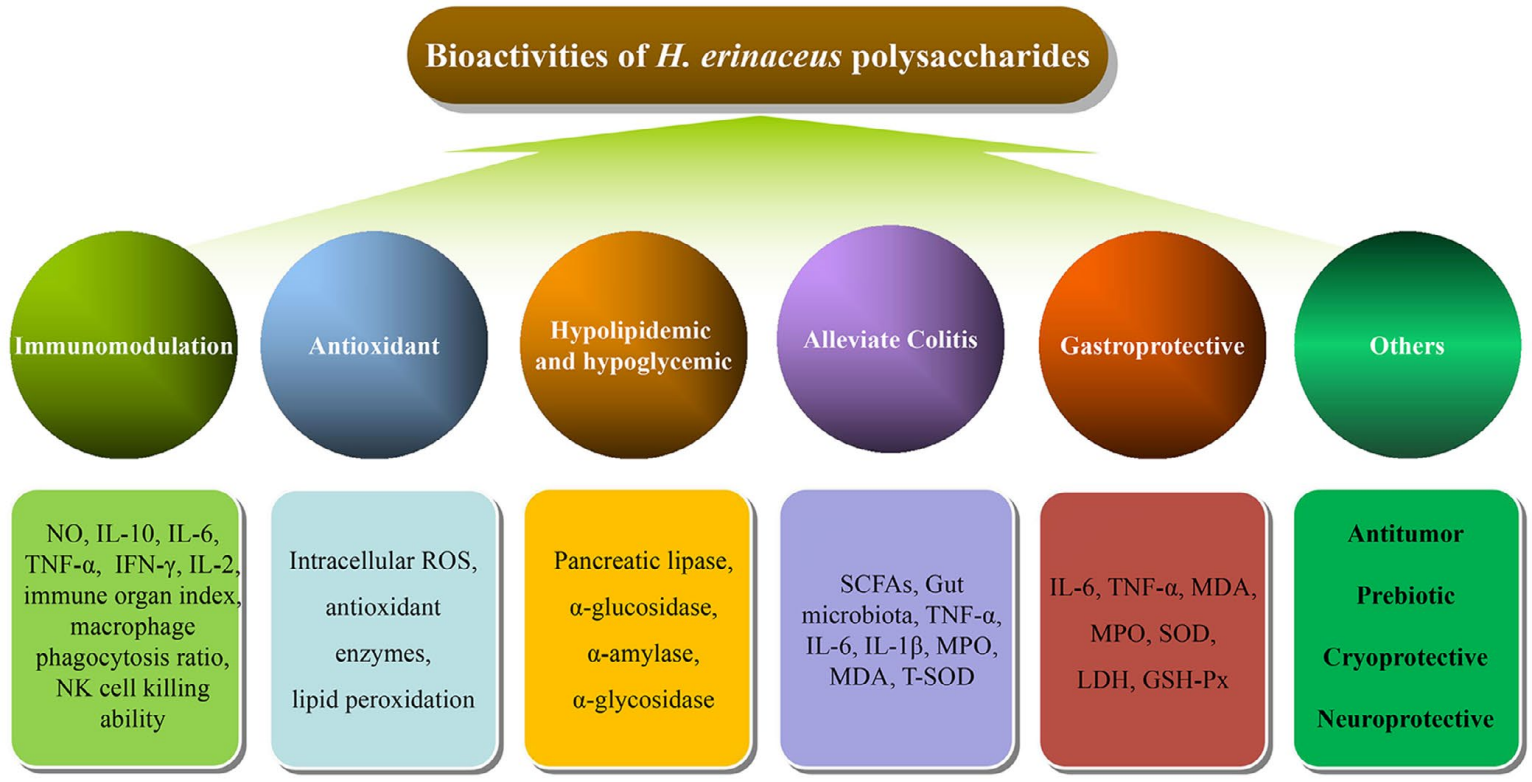
Bioactivities of *H. erinaceus* polysaccharides.

The *H. erinaceus* polysaccharides exhibit a range of bioactivities, including immunomodulation activities (Chaiyasut et al. 2018; Han et al. 2023; Li et al. 2016; Liu et al. 2021; Ren, Sun, et al. 2023; Wu and Huang 2021; Wu et al. 2017, 2018; Yang et al. 2022), antioxidant properties (Chen et al. 2015; Han et al. 2013; Liao et al. 2020; Qin et al. 2020; Su et al. 2023; Tu et al. 2021; Yan et al. 2018; Zhuang et al. 2023), hypolipidemic activities, hypoglycemic effects (Cui et al. 2023; Su et al. 2023; Yan et al. 2018), alleviation of colitis (Ren, Sun, et al. 2023; Ren, Xu, et al. 2023; Wang et al. 2018), gastroprotective effects (Hou et al. 2022; Wang et al. 2022), antitumor properties (Chen et al. 2015; Qin et al. 2017), cryoprotective effects (Shirokikh et al. 2020), neuroprotective properties (Cheng et al. 2016), prebiotic activities (Zhuang et al. 2023) and so on (Figure 3 and Table 3).

**TABLE 3:** The bioactivities and underlying mechanisms associated with the polysaccharides derived from *Hericium erinaceus*.

Name	Activity	Target	Dose	Action or mechanism	References
BG	Immunomodulation	BALB/cMlac mice	100, 150, and 200 mg/kg	Stimulated the expression of cytokines and increased antioxidant enzymes levels	(Chaiyasut et al. 2018)
HEP‐1	Immunomodulation	RAW264.7	50, 100, 200 and 400 μg/mL	Markedly promoted the production of NO, cytokines, in addition to promoting the phosphorylation of various signaling molecules	(Yang et al. 2022)
HEP‐S	Immunomodulation	RAW264.7; Balb/c mice	100–1000 μg/mL	Increased pinocytosis and phagocytosis, enhanced secretion of NO and cytokines, and promoted B and T lymphocyte proliferation	(Wu et al. 2018)
HEP‐W	Immunomodulation	RAW264.7	100–1000 μg/mL	Increased phagocytosis and pinocytosis, and enhanced secretion of NO and cytokines	(Wu et al. 2017)
HPB‐3	Immunomodulation	RAW264.7	50–800 μg/mL	Stimulated the production of NO	(Li et al. 2016)
EHEP	Immunomodulation	RAW264.7; Balb/c mice	0.391–25 μg/mL; 125, 250, and 500 mg/kg	Promoted phagocytosis, NO production, and expression of CD86 and CD40; Activated peritoneal macrophages and improved their immunomodulation capabilities	(Liu et al. 2021)
HEP	Immunomodulation	Kunming mice	80, 160, and 320 mg/kg	Enhanced the microstructural integrity, immune response, antioxidant capacity, and apoptotic processes within the thymus and spleen	(Han et al. 2023)
HEP‐W	Immunomodulation	Balb/c mice	75, 150, and 300 mg/kg	Enhanced immune organ index, macrophage phagocytosis ratio, natural killer cell killing ability, interleukin‐2 production and splenocyte proliferation	(Wu and Huang 2021)
HEP10	Immunomodulation; Alleviate Colitis	RAW264.7; C57BL/6 mice	25–200 μg/mL 50, 100, and 200 mg/kg	Inhibited production of cytokines, COX‐2 and iNOS; Suppressed NLRP3 inflammasome activation	(Ren, Sun, et al. 2023)
FHEP; HEP	Antioxidant; Hypoglycemic and hypolipidemic	N/A	10 mg/mL	FHEP exhibited higher antioxidant, hypoglycemic and hypolipidemic activities than HEP	(Su et al. 2023)
HEP	Antioxidant	IPEC‐J2	3.91–62.5 μg/mL	Safeguarded IPEC‐J2 from DON‐induced oxidative stress, inhibited apoptosis in these cells, and diminished ROS production	(Qin et al. 2020)
HEP	Antioxidant	Wistar rats	300 mg/kg	Safeguard renal function by declining levels of lipid peroxidation and enhancing activity of antioxidant enzymes	(Han et al. 2013)
HEP_N_	Antioxidant	GES‐1	125, 250, and 500 μg/mL	Decreased ROS levels, promoted proliferation and inhibited necrosis of GES‐1 cells, regulated potential and maintained permeability of mitochondrial membrane	(Liao et al. 2020)
HEP	Antioxidant; Antitumor	N/A; MCF‐7 and HeLa	50, 100, and 200 μg/mL	Scavenged DPPH and hydroxyl radicals; Inhibited growth of MCF‐7 and HeLa cells	(Chen et al. 2015)
HEP	Antioxidant	N/A	0.5–3 mg/mL	HEP had the capacity to scavenge radicals	(Tu et al. 2021)
HEP‐W/S/C/A	Antioxidant; Hypoglycemic	N/A	0.5–3 mg/mL	All HEPs had radicals scavenging capacity and ferric reducing activity; All polysaccharides exhibited hypoglycemic activity, and HEP‐C had the most pronounced inhibitory effects on both α‐amylase and α‐glycosidase	(Yan et al. 2018)
HEP‐W; HEP‐A	Antioxidant; Prebiotic	N/A	1–3 mg/mL	Had radicals scavenging capacity; Decreased the pH value, regulated microbiota composition, and increased the content of gas and SCFA	(Zhuang et al. 2023)
HEP‐1	Hypoglycemic; hypolipidemic	Kunming mice	400 mg/kg	Facilitated the uptake of serum glucose; Reduced hepatic lipid accumulation and inhibited fatty acid synthesis	(Cui et al. 2023)
HECP; HERP	Alleviate Colitis	Kunming mice	100, 200, and 400 mg/kg	Augment SCFAs production, reduced pH values and improved moisture amounts	(Wang et al. 2018)
HEMP	Alleviate Colitis	Cynomolgus monkeys	500 mg/kg	Alleviated the pathologies of UC, enhanced nutritional status, decreased diarrhea frequency, reduced inflammation, and altered the gut microbiota composition	(Ren, Xu, et al. 2023)
HEP; RP‐S	Gastroprotective	SD rats; GES‐1	100, 200, and 400 mg/kg; 10–160 μg/mL	Decreased the levels of pro‐inflammatory cytokines and MDA, reduced activity of MPO, and improved release of gastric defensive factors and SOD activity; RP‐S treatment led to an increase in cell viability, LDH activity, and MDA content, while concurrently decreasing GSH‐Px and SOD activities	(Wang et al. 2022)
HMP; HFP	Gastroprotective	SD rats; GES‐1	19.8 mg/kg; 25–1000 μg/mL	HMP had better gastric protective activity than HFP; Promoted proliferation and migration, and changed biochemical indices of GES‐1 cells	(Hou et al. 2022)
HEP‐2	Antitumor	HeLa	0.1–1.6 mg/mL	Inhibited the growth of HeLa cells	(Qin et al. 2017)
PF	Cryoprotective	Human venous blood	1%–0.1%	Ensured viability when stored at temperatures of −80°C and −20°C	(Shirokikh et al. 2020)
HEPS	Neuroprotective	PC12	25–250 μg/mL	Alleviated amyloid beta (Aβ)‐induced neurotoxicity	(Cheng et al. 2016)

Abbreviation: N/A: not available.

### 4.1 Immunomodulation Activity

Studies have demonstrated that fungal polysaccharides interact with various pattern recognition receptors (PRRs) located on the cell surface, including dendritic cell‐associated C‐type lectin‐1 (Dectin‐1), Toll‐like receptors (TLRs), etc. The diverse origins and structural characteristics of these polysaccharides enable them to bind to distinct receptors, which in turn activate different immune cell populations. Consequently, polysaccharides can enhance cellular immune responses, modulate cytokine secretion, and exert immunomodulatory effects (Deng et al. 2018; Li et al. 2017).

**FIGURE 4:**
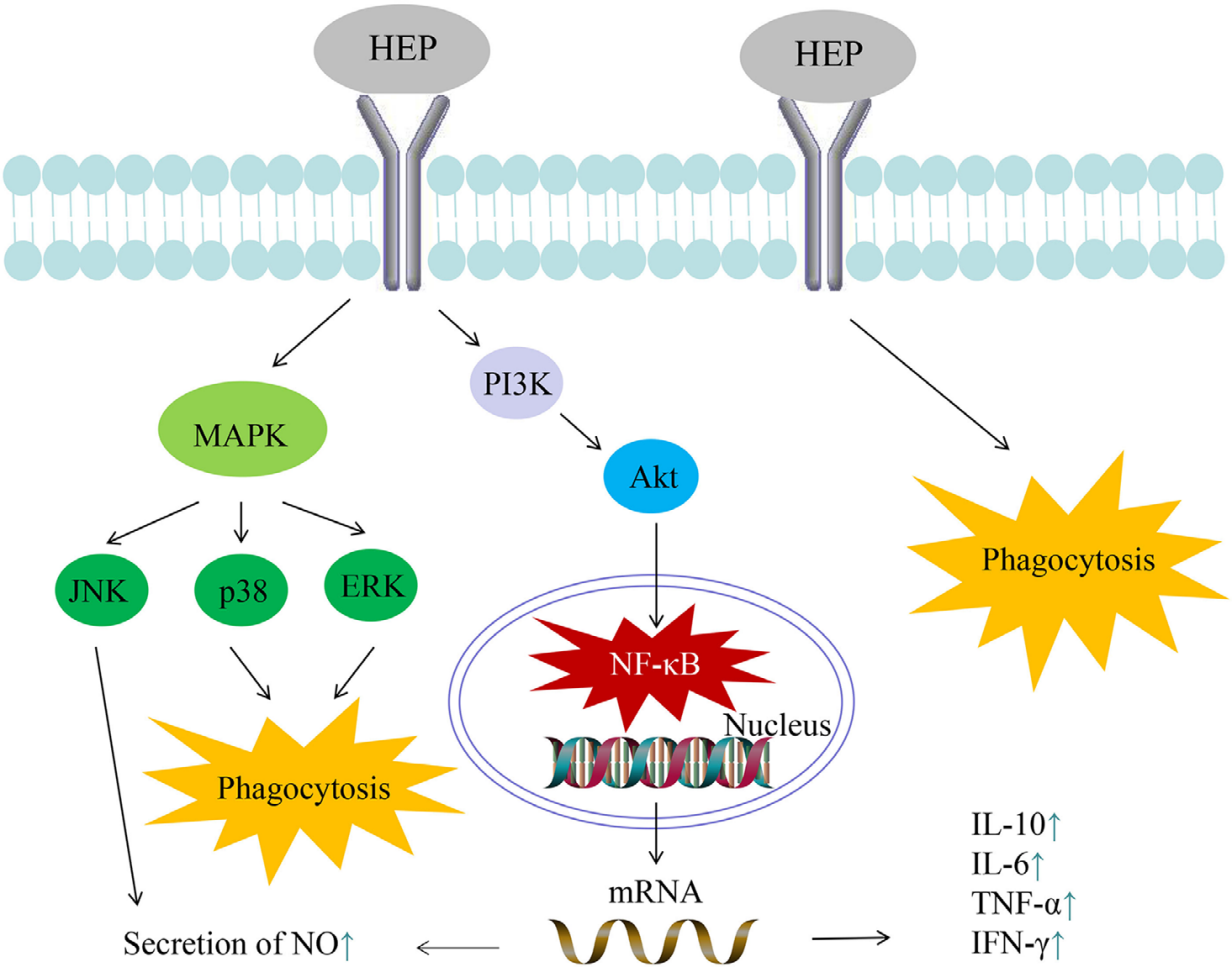
The potential immunomodulation mechanism of HEP in RAW264.7 cells (Yang et al. 2022).

Yang et al. (2022) demonstrated that the polysaccharide HEP‐1 derived from *H. erinaceus* exhibited notable immunomodulation effects in RAW264.7 cells. The experimental findings demonstrated that HEP‐1 markedly enhanced the production of nitric oxide (NO), interleukins 10 (IL‐10), interleukins 6 (IL‐6), tumor necrosis factor‐alpha (TNF‐α) and interferon‐gamma (IFN‐γ), in addition to promoting the phosphorylation of various signaling molecules. They speculated that HEP‐1 increased immune responses via MAPK, PI3K/Akt and NF‐κB signaling pathways (Figure 4). Wu and Huang (2021) established mouse models of immunosuppression induced by cyclophosphamide (CTX), and then they gavaged the mice with polysaccharides. The experimental results indicated that HEP‐W had the potential to markedly improve various immunological parameters, including the immune organ index, macrophage phagocytosis ratio, natural killer (NK) cell killing ability, interleukin‐2 (IL‐2) production, and splenocyte proliferation. Liu et al. (2021) successfully obtained an enzymatic hydrolysis polysaccharide, designated as EHEP, with remarkable immunomodulation activity. In RAW264.7 cells, EHEP markedly promoted the phagocytosis, NO production, and expression of CD86 and CD40 costimulatory molecules. In vivo, EHEP had the potential to activate peritoneal macrophages and promote their immunomodulation capabilities.

In vitro, HEP interacted with receptors on the cell membrane of RAW264.7 cells, resulting in the phosphorylation of JNK, p38, and ERK, thereby activating the MAPK signaling pathway. Specifically, JNK facilitated the secretion of NO, while p38 and ERK contributed to the induction of phagocytosis. Additionally, the NF‐κB pathway functioned as a transcription factor within the cell nucleus, directly modulating the expression of various inflammatory cytokines, including IL‐10, IL‐6, TNF‐α, and IFN‐γ (Chen et al. 2024; Liu et al. 2025; Yang et al. 2022). In vivo, HEP was demonstrated to enhance various immunological parameters in mice, including the activity of immune cells and the function of immune organs, thereby contributing to the overall improvement of immune function in these animals (Liu et al. 2021; Wu and Huang 2021). While experimental findings from animal models investigating HEP have demonstrated their potential to enhance immune function, these results do not comprehensively reflect their effects on the human immune system. Therefore, further research is necessary, including the development of humanized animal models and clinical trials, to substantiate the influence of HEP in humans.

### 4.2 Antioxidant Activity

Qin et al. (2020) conducted a study in which they extracted *H. erinaceus* polysaccharides and investigated the protective effects of these polysaccharides, referred to as HEP, against oxidative stress induced by deoxynivalenol (DON). Their findings indicated that HEP effectively safeguarded IPEC‐J2 cells from oxidative stress induced by DON, diminished intracellular ROS production and inhibited apoptosis of these cells. Liao et al. (2020) established oxidative damage models of GES‐1 cells induced by hydrogen peroxide (H_2_O_2_) and studied the protective effects of HEP_N_ in relation to oxidative damage. The findings indicated that the HEP_N_ could decrease the levels of ROS, promote proliferation and inhibit necrosis of GES‐1 cells, regulate potential and maintain the permeability of the mitochondrial membrane. Han et al. (2013) gavaged Wistar rats with *H. erinaceus* polysaccharides (300 mg/kg, 15 days) and evaluated the in vivo antioxidant activity of HEP. Results found that compared to the renal ischemia–reperfusion group, the group that received pre‐administration of HEP demonstrated a reduction in blood urea nitrogen and serum creatinine levels, as well as an enhancement in creatinine clearance. In addition, renal ischemia reperfusion could result in renal oxidative injury damage, and HEP had the potential to safeguard renal function by enhancing the activities of antioxidant enzymes and declining levels of lipid peroxidation.

### 4.3 Hypolipidemic and Hypoglycemic Activities

Su et al. (2023) isolated two distinct polysaccharides from *H. erinaceus* : the polysaccharides degraded by *Lactobacillus gasseri* JM1 (designated as FHEP) and the undegraded polysaccharides (referred to as HEP). The researchers subsequently evaluated their hypolipidemic and hypoglycemic effects. The findings indicated that FHEP and HEP effectively reduced hyperlipidemia and hyperglycemia via inhibiting enzymatic activities of pancreatic lipase, α‐glucosidase and α‐amylase. Notably, the hypoglycemic and hypolipidemic effects of FHEP were found to be superior to those of HEP. Yan et al. (2018) extracted four types of *H. erinaceus* polysaccharides utilizing various extraction methods: hot water (HEP‐W), sodium chloride (HEP‐S), citric acid (HEP‐C), and alkaline solution (HEP‐A). All polysaccharides exhibited hypoglycemic activity, and HEP‐C had the most pronounced inhibitory effects against both α‐glucosidase and α‐amylase. Cui et al. (2023) reported that HEP‐1 had excellent hypoglycemic and hypolipidemic activities. The researchers investigated the hypoglycemic and hypolipidemic mechanisms of HEP‐1 using mouse models of type 2 diabetes mellitus (T2DM). Their findings demonstrated that HEP‐1 facilitated the uptake of serum glucose by enhancing hepatic glycogen synthesis via activating IRS/PI3K/AKT signaling pathways. Additionally, HEP‐1 was shown to reduce hepatic lipid accumulation and inhibit fatty acid synthesis through activating the AMPK/SREBP‐1c signaling pathway. Consequently, HEP‐1 appeared to be instrumental in mitigating the metabolic imbalances in glucose and lipid metabolism associated with T2DM.

### 4.4 Alleviate Colitis

Ren, Sun, et al. (2023) reported a low weight *H. erinaceus* polysaccharide HEP10 with the activity of alleviating colitis in a dextran sulfate sodium (DSS) induced mice model. The tests demonstrated that HEP10 had been shown to mitigate oxidative damage and cytokine production, suppress NLRP3 inflammasome activation, and impede the phosphorylation of NF‐κB p65, Akt, and MAPK. Additionally, it appeared to modulate both structure and metabolism in gut microbiota. Wang et al. (2018) explored the impact of polysaccharides on the colonic health of murine subjects. The results suggested that the polysaccharides (HECP and HERP) treated groups had the potential to augment short‐chain fatty acids (SCFAs) production in the contents of the colon and cecum, as well as in fecal matter. Additionally, a notable decrease in pH values was recorded, while the moisture amounts were significantly improved in the HERP‐H group (400 mg/kg bw). Ren, Xu, et al. (2023) obtained *H. erinaceus* polysaccharides from mycelium and investigated their potential therapeutic effects on ulcerative colitis (UC) in cynomolgus monkeys. The study revealed that the polysaccharides could significantly alleviate the pathologies associated with UC, improve nutritional status, decrease diarrhea frequency, reduce inflammation, and modify gut microbiota composition in affected monkeys.

**FIGURE 5:**
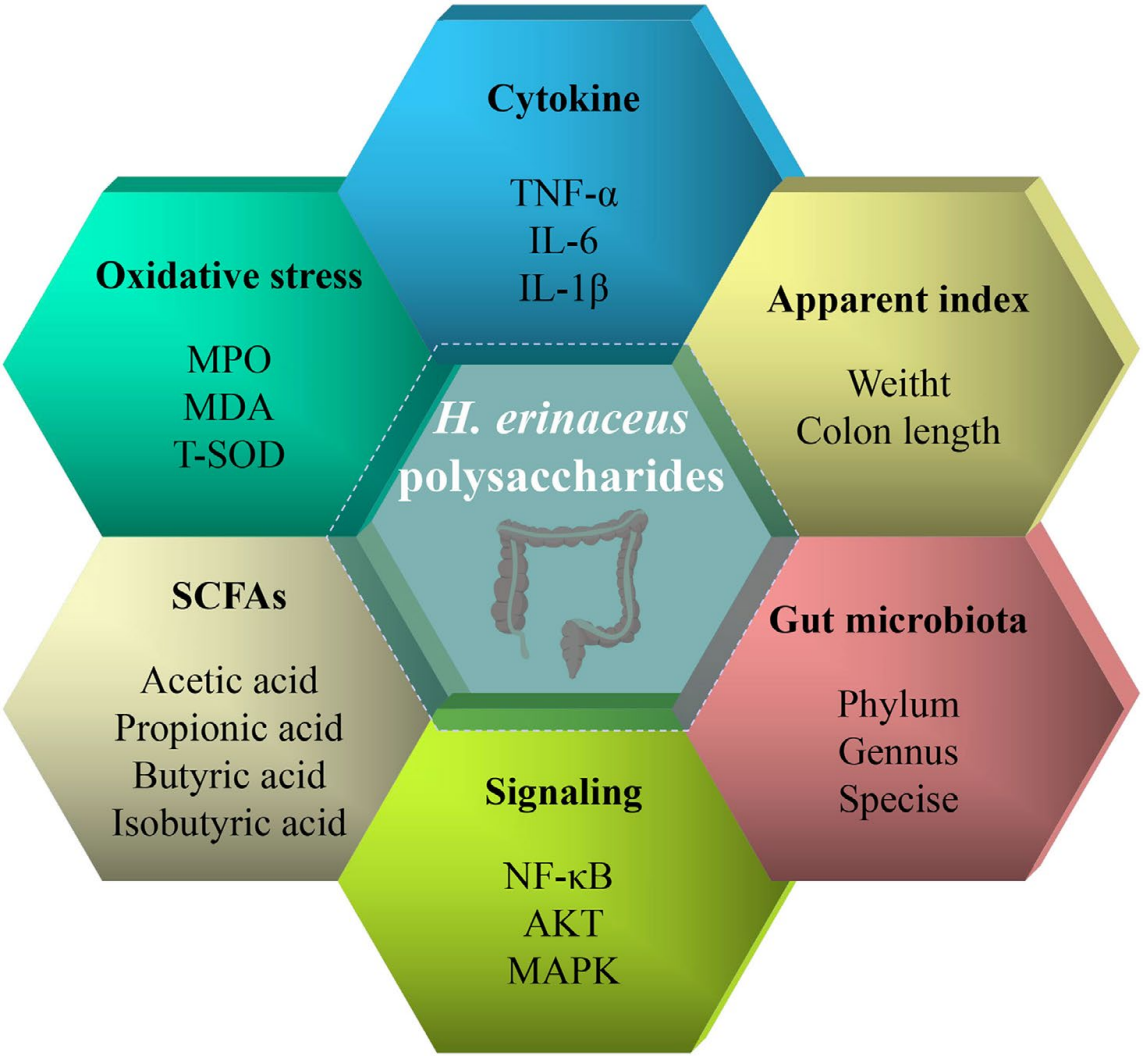
The potential mechanism of HEP in alleviating colitis.

The proposed mechanism by which HEP alleviates colitis is illustrated in Figure 5. HEP can alleviate colitis by various mechanisms, including modulating cytokines, enhancing apparent index, balancing gut microbiota, influencing signaling pathways, regulating SCFAs, and reducing oxidative stress. Specifically, (1) HEP suppresses the inflammatory response by influencing cytokines like TNF‐α, IL‐6, and IL‐1β; (2) HEP positively influences apparent index like weight and colon length, helping to preserve the organism's normal physiological condition; (3) HEP acts on the phylum, genus, and species levels of gut microbiota, maintaining the balance of the intestinal microecological environment; (4) HEP affects signaling pathways like NF‐κB, AKT, MAPK, modulating the inflammatory response at the molecular level; (5) HEP raises the levels of SCFAs, including acetic acid, propionic acid, butyric acid, and isobutyric acid to modulate intestinal immunity; (6) HEP enhances the body's antioxidant capacity by affecting indicators such as MPO, MDA, and T‐SOD, thereby reducing oxidative stress damage to colon tissues (Ren, Sun, et al. 2023; Ren, Xu, et al. 2023; Wang et al. 2018). In summary, HEP modulates the aforementioned pathways to effectively alleviate colitis.

### 4.5 Gastroprotective Activity

Wang et al. (2022) investigated the gastroprotective properties of HEP using a rat model of acetic acid‐induced gastric ulcers (GU) and a hydrogen peroxide (H_2_O_2_)‐induced injury model in GES‐1 cells. The findings from the animal studies indicated that HEP administration resulted in a reduction in pro‐inflammatory cytokine levels, such as IL‐6 and TNF‐α, as well as a decrease in malondialdehyde (MDA) level and myeloperoxidase (MPO) activity. Furthermore, HEP treatment was associated with an enhancement in the release of gastric protective factors, along with an increase in superoxide dismutase (SOD) activity within gastric tissues of GU rats. Subsequently, the researchers screened bioactive components of purified HEP, identifying RP‐S as exhibiting notable gastroprotective effects in the H_2_O_2_‐induced injury model in GES‐1 cells. Cellular experiments revealed that RP‐S treatment increased cell viability, lactate dehydrogenase (LDH) activity, and MDA content, while concurrently decreasing the activities of SOD and glutathione peroxidase (GSH‐Px) in GES‐1 cells. Hou et al. (2022) compared the gastric protective effects of two polysaccharides: HFP (source: fruiting body) and HMP (source: mycelium). The findings demonstrated that both polysaccharides exhibited protective properties against acute gastric ulcers induced by ethanol in rats, and HMP had better gastric protective activity than HFP. In addition, HMP and HFP could promote proliferation and migration, and change biochemical indices of GES‐1 cells to resist alcohol‐induced gastric mucosal injury.

### 4.6 Others

In addition to the above biological activities, *H. erinaceus* polysaccharides had other bioactivities. Chen et al. (2015) indicated that *H. erinaceus* polysaccharides could inhibit the proliferation of MCF‐7 and HeLa cells. Similarly, Qin et al. (2017) discovered that the purified fraction HEP‐2 could inhibit the growth of HeLa cells. Shirokikh et al. (2020) proved that PF supplements could ensure the viability of human venous blood when stored at −80°C and −20°C. Cheng et al. (2016) demonstrated that HEPS had the potential to alleviate neurotoxicity induced by amyloid beta (Aβ) in PC12 cells. Zhuang et al. (2023) isolated two polysaccharides from *H. erinaceus* , which exhibited prebiotic properties. Both polysaccharides were found to decrease the pH value, enhance the production of gas and SCFAs, and regulate microbiota composition during fermentation.

Current research findings indicate a diverse range of biological activities associated with polysaccharides derived from *H. erinaceus* . These polysaccharides exhibit properties that contribute to the enhancement of the immune system, the protection of gastrointestinal health, the provision of antioxidant effects, and so forth (Ge et al. 2025; Hou et al. 2020). Nevertheless, the majority of investigations have predominantly focused on in vitro chemical assays, cellular studies, and in vivo animal experiments. To advance the understanding of the functional properties of polysaccharides from *H. erinaceus* , the implementation of clinical trials is essential. Furthermore, the polysaccharide‐enriched extracts from *H. erinaceus* may contain bioactive small molecules, including erinacine and erinacoside. These compounds have the potential to influence the biological activity of polysaccharides to a certain degree, and subsequent research may be conducted in relation to this topic (Qiu et al. 2024).

## 5 Conclusions and Perspectives

*H. erinaceus* is a fungus that possesses both medicinal and nutritional properties. It encompasses a diverse array of bioactive constituents, including polysaccharides, erinacines, hericenones, proteins, terpenes, ergosterol, peptides, and so forth. Among them, *H. erinaceus* polysaccharides have attracted the attention of scientists because of their excellent pharmaceutical value. This study primarily examines the extraction techniques, purification processes, structure and bioactivities of *H. erinaceus* polysaccharides.

Firstly, we summarize the methodologies employed for the extraction and purification of *H. erinaceus* polysaccharides. The predominant technique utilized for extraction is hot water extraction. Furthermore, alternative extraction methods are also discussed, including different solvent extractions (saline, acid, alkali), microwave‐assisted extraction, enzymatic hydrolysis, ultrasound‐assisted enzymatic extraction, supercritical CO_2_ extraction, and non‐isothermal autohydrolysis. The purification methods of *H. erinaceus* polysaccharides are column chromatography, gradual ethanol precipitation, membrane filtration, and so forth. The emerging technologies facilitate the separation of *H. erinaceus* polysaccharides in a manner that is more efficient, environmentally sustainable, and cost‐effective.

Secondly, we conduct an analysis of the structure of *H. erinaceus* polysaccharides, encompassing the molecular weight, monosaccharide composition, and distinctive structural properties. The structural complexity of polysaccharides presents significant challenges for accurate analysis using traditional methodologies. To thoroughly elucidate the intricate structure—such as monosaccharide composition, types of glycosidic linkages, branching patterns, molecular weight distribution, and advanced spatial conformations—it is essential to employ advanced analytical technologies. In the future, it may be feasible to utilize machine learning techniques to analyze the fundamental structure of *H. erinaceus* polysaccharides.

Thirdly, we generalize the bioactivities and potential mechanisms of action associated with polysaccharides derived from *H. erinaceus* , such as immunomodulation activities, antioxidant properties, hypoglycemic activities, hypolipidemic effects, the alleviation of colitis, gastroprotective effects, antitumor properties, cryoprotective effects, neuroprotective properties, and prebiotic activities, among others. In summary, *H. erinaceus* polysaccharides exhibit significant potential for the treatment of disorders affecting the digestive system, improving immunity, reducing blood sugar, anti‐oxidation, and so forth.

In recent years, notable progress has been achieved in the investigation of *H. erinaceus* polysaccharides. Nonetheless, the current research exhibits several notable limitations. Firstly, the research models predominantly rely on in vitro cell cultures or animal studies, with a paucity of clinical trials to validate findings in human subjects. Secondly, the underlying mechanisms of action remain inadequately defined. Most investigations have been confined to phenotypic observations without elucidating the molecular pathways. Thirdly, there is a lack of systematic studies addressing the structure–activity relationship (SAR) of *H. erinaceus* polysaccharides, despite the recognized influence of molecular structure on biological activity. In light of these challenges, several recommendations are proposed for future research directions. Primarily, there is a need to advance clinical studies and facilitate clinical translation. Additionally, innovation in delivery technologies should be pursued, including the exploration of nanotechnology‐based delivery systems for *H. erinaceus* polysaccharides. Finally, structure–activity relationship (SAR) modeling is employed to investigate the correlation between their structure and biological activity, and to develop a comprehensive database for *H. erinaceus* polysaccharides.

## Author Contributions

**Naxin Sun:** writing – review and editing; **Chao Li:** supervision; **Wu Liang:** conceptualization; **Zijian Wu:** supervision; **Xuemei Han:** conceptualization; **Suyun Xu:** conceptualization.

## Conflicts of Interest

The authors declare no conflicts of interest.

## Data Availability

Data sharing not applicable to this article as no datasets were generated or analyzed during the current study.

## References

[fsn371275-bib-0001] Atmaca, H. , Ç. Pulat , S. Ilhan , and F. Kalyoncu . 2024. “ *Hericium erinaceus* Extract Induces Apoptosis via PI3K/AKT and RAS/MAPK Signaling Pathways in Prostate Cancer Cells.” Chemistry & Biodiversity 21, no. 12: 1–9. 10.1002/cbdv.202400905. 39183463

[fsn371275-bib-0002] Calabretti, A. , S. M. Mang , A. Becce , et al. 2021. “Comparison of Bioactive Substances Content Between Commercial and Wild‐Type Isolates of *Pleurotus eryngii* .” Sustainability 13, no. 7: 1–12. 10.3390/su13073777.

[fsn371275-bib-0003] Chaiyasut, C. , N. Pengkumsri , B. S. Sivamaruthi , et al. 2018. “Extraction of β‐Glucan of *Hericium erinaceus*, *Avena sativa* L., and *Saccharomyces cerevisiae* and In Vivo Evaluation of Their Immunomodulatory Effects.” Food Science and Technology 38: 138–146. 10.1590/fst.18217.

[fsn371275-bib-0004] Chen, P. Y. , Y. Y. Yong , Y. F. Gu , Z. L. Wang , S. Z. Zhang , and L. Lu . 2015. “Comparison of Antioxidant and Antiproliferation Activities of Polysaccharides From Eight Species of Medicinal Mushrooms.” International Journal of Medicinal Mushrooms 17, no. 3: 287–295. 10.1615/IntJMedMushrooms.v17.i3.80. 25954912

[fsn371275-bib-0005] Chen, S. K. , Y. H. Li , X. Wang , et al. 2023. “Evaluation of the ‘Relative Ordered Structure of *Hericium erinaceus* Polysaccharide’ From Different Origins: Based on Similarity and Dissimilarity.” Journal of Agricultural and Food Chemistry 71, no. 46: 17886–17898. 10.1021/acs.jafc.3c04329. 37955257

[fsn371275-bib-0006] Chen, Y. , M. Tao , X. Wu , et al. 2024. “Current Status and Research Progress of Oncolytic Virus.” Pharmaceutical Science Advances 2: 1–13. 10.1016/j.pscia.2024.100037. PMC12710064 41550155

[fsn371275-bib-0007] Cheng, J. H. , C. L. Tsai , Y. Y. Lien , M. S. Lee , and S. C. Sheu . 2016. “High Molecular Weight of Polysaccharides From *Hericium erinaceus* Against Amyloid Beta‐Induced Neurotoxicity.” BMC Complementary and Alternative Medicine 16: 1–9. 10.1186/s12906-016-1154-5. 27266872 PMC4895996

[fsn371275-bib-0008] Cong, B. 2024. “Perspectives in Food & Medicine Homology.” Journal of Food and Medicinal Homology 1, no. 1: 1. 10.26599/FMH.2024.9420018.

[fsn371275-bib-0009] Cui, W. J. , X. L. Song , X. P. Li , L. Jia , and C. Zhang . 2023. “Structural Characterization of *Hericium erinaceus* Polysaccharides and the Mechanism of Anti‐T2DM by Modulating the Gut Microbiota and Metabolites.” International Journal of Biological Macromolecules 242: 1–11. 10.1016/j.ijbiomac.2023.125165. 37270132

[fsn371275-bib-0010] Deng, C. , H. T. Fu , J. Y. Shang , J. H. Chen , and X. Xu . 2018. “Dectin‐1 Mediates the Immunoenhancement Effect of the Polysaccharide From *Dictyophora indusiata* .” International Journal of Biological Macromolecules 109: 369–374. 10.1016/j.ijbiomac.2017.12.113. 29274416

[fsn371275-bib-0011] Deng, Y. , J. Zhao , and S. P. Li . 2023. “Quantitative Estimation of Enzymatic Released Specific Oligosaccharides From *Hericium erinaceus* Polysaccharides Using CE‐LIF.” Journal of Pharmaceutical Analysis 13, no. 2: 201–208. 10.1016/j.jpha.2022.11.004. 36908854 PMC9999295

[fsn371275-bib-0012] Dong, W. J. , Y. C. Li , S. R. Xue , et al. 2024. “Yeast Polysaccharides: The Environmentally Friendly Polysaccharides With Broad Application Potentials.” Comprehensive Reviews in Food Science and Food Safety 23, no. 5: 1–22. 10.1111/1541-4337.70003. 39223755

[fsn371275-bib-0013] El‐Ramady, H. , N. Abdalla , K. Badgar , et al. 2022. “Edible Mushrooms for Sustainable and Healthy Human Food: Nutritional and Medicinal Attributes.” Sustainability 14, no. 9: 1–30. 10.3390/su14094941.

[fsn371275-bib-0014] Ezurike, P. U. , E. Odunola , T. A. Oke , et al. 2023. “ *Ganoderma lucidum* Ethanol Extract Promotes Weight Loss and Improves Depressive‐Like Behaviors in Male and Female Swiss Mice.” Physiology & Behavior 265: 1–11. 10.1016/j.physbeh.2023.114155. 36907499

[fsn371275-bib-0015] Ferreira, I. , J. A. Vaz , M. H. Vasconcelos , and A. Martins . 2010. “Compounds From Wild Mushrooms With Antitumor Potential.” Anti‐Cancer Agents in Medicinal Chemistry 10, no. 5: 424–436. 10.2174/1871520611009050424. 20545620

[fsn371275-bib-0016] Fu, G.‐Q. , Y.‐X. Li , Y. He , H. Zhang , and X. Ma . 2025. “Extraction, Structure and Bioactivity of Tremella Fuciformis Polysaccharides: A Review.” 2, no. 4: 1–9. 10.26599/FMH.2025.9420038.

[fsn371275-bib-0017] Ganesan, K. , and B. J. Xu . 2018. “Anti‐Obesity Effects of Medicinal and Edible Mushrooms.” Molecules 23, no. 11: 1–26. 10.3390/molecules23112880. PMC6278646 30400600

[fsn371275-bib-0018] Ge, F. Z. , Y. Chen , B. S. Wang , W. X. Zhou , B. X. Du , and L. Hou . 2025. “Bioactive Polysaccharides From *Hericium erinaceus*: Extraction, Structure, Bioactivities, and Applications.” Molecules 30, no. 8: 1–39. 10.3390/molecules30081850. PMC12029305 40333844

[fsn371275-bib-0019] Gong, P. , S. Yue , J. Wang , et al. 2025. “Effect of Ultrasound Synergistic pH Shift Modification Treatment on *Hericium erinaceus* Protein Structure and Its Application in 3D Printing.” International Journal of Biological Macromolecules 295: 1–13. 10.1016/j.ijbiomac.2025.139562. 39788265

[fsn371275-bib-0020] Gu, H. F. , L. Liang , Y. N. Kang , R. M. Yu , J. H. Wang , and D. Fan . 2024. “Preparation, Characterization, and Property Evaluation of *Hericium erinaceus* Peptide‐Calcium Chelate.” Frontiers in Nutrition 10: 1337407. 10.3389/fnut.2023.1337407. 38264190 PMC10803561

[fsn371275-bib-0021] Han, Y. J. , J. L. Huang , C. F. Zhao , et al. 2023. “ *Hericium erinaceus* Polysaccharide Improves the Microstructure, Immune Function, Proliferation and Reduces Apoptosis of Thymus and Spleen Tissue Cells of Immunosuppressed Mice.” Bioscience, Biotechnology, and Biochemistry 87, no. 3: 279–289. 10.1093/bbb/zbac198. 36494196

[fsn371275-bib-0022] Han, Z. H. , J. M. Ye , and G. F. Wang . 2013. “Evaluation of In Vivo Antioxidant Activity of *Hericium erinaceus* Polysaccharides.” International Journal of Biological Macromolecules 52: 66–71. 10.1016/j.ijbiomac.2012.09.009. 23000690

[fsn371275-bib-0023] He, H. Q. , C. Liu , C. S. Shao , Y. H. Wu , and Q. Huang . 2022. “Green Synthesis of Ultrasmall Selenium Nanoparticles (SeNPs) Using *Hericium erinaceus* Polysaccharide (HEP) as Nanozymes for Efficient Intracellular Antioxidation.” Materials Letters 317: 1–4. 10.1016/j.matlet.2022.132079.

[fsn371275-bib-0024] He, X. R. , X. X. Wang , J. C. Fang , et al. 2017. “Structures, Biological Activities, and Industrial Applications of the Polysaccharides From *Hericium erinaceus* (Lion's Mane) Mushroom: A Review.” International Journal of Biological Macromolecules 97: 228–237. 10.1016/j.ijbiomac.2017.01.040. 28087447

[fsn371275-bib-0025] Hoon, K. , J. Jae‐Hyeon , H. Jong‐Hyun , J. Heon‐Sang , L. Hyeon‐Yong , and Y. Kwang‐Won . 2010. “Enhancement of Immunostimulation and Anti‐Metastasis in Submerged Culture of Bearded Tooth Mushroom (*Hericium erinaceum*) Mycelia by Addition of Ginseng Extract.” Food Science and Biotechnology 19, no. 5: 1259–1266.

[fsn371275-bib-0026] Hou, C. L. , L. Y. Liu , J. Y. Ren , M. Huang , and E. D. Yuan . 2022. “Structural Characterization of Two *Hericium erinaceus* Polysaccharides and Their Protective Effects on the Alcohol‐Induced Gastric Mucosal Injury.” Food Chemistry 375: 1–8. 10.1016/j.foodchem.2021.131896. 34954576

[fsn371275-bib-0027] Hou, C. Y. , L. L. Chen , L. Z. Yang , and X. L. Ji . 2020. “An Insight Into Anti‐Inflammatory Effects of Natural Polysaccharides.” International Journal of Biological Macromolecules 153: 248–255. 10.1016/j.ijbiomac.2020.02.315. 32114173

[fsn371275-bib-0028] Iqbal, T. , M. Sohaib , S. Iqbal , and H. Rehman . 2024. “Comprehensive Nutritional Profiling and Antioxidant Capacity Assessment of Indigenous Mushrooms *Pleurotus ostreatus* and *Agaricus bisporus* .” Czech Journal of Food Sciences 42, no. 3: 174–183. 10.17221/158/2023-cjfs.

[fsn371275-bib-0029] Ji, X. L. , C. Y. Hou , Y. Z. Yan , M. M. Shi , and Y. Q. Liu . 2020. “Comparison of Structural Characterization and Antioxidant Activity of Polysaccharides From Jujube (*Ziziphus jujuba* Mill.) Fruit.” International Journal of Biological Macromolecules 149: 1008–1018. 10.1016/j.ijbiomac.2020.02.018. 32032709

[fsn371275-bib-0030] Ji, X. L. , M. S. Yin , H. Nie , and Y. Q. Liu . 2020. “A Review of Isolation, Chemical Properties, and Bioactivities of Polysaccharides From *Bletilla striata* .” BioMed Research International 2020, no. 1: 5391379. 10.1155/2020/5391379. 32596325 PMC7273373

[fsn371275-bib-0031] Jia, L.‐F. , P. Chen , G.‐D. Qu , et al. 2025. “A Comprehensive Review of the Sedative‐Hypnotic Mechanisms of Edible Fungi.” 2, no. 4: 1–9. 10.26599/FMH.2025.9420049.

[fsn371275-bib-0032] Li, Q. Z. , D. Wu , S. Zhou , et al. 2016. “Structure Elucidation of a Bioactive Polysaccharide From Fruiting Bodies of *Hericium erinaceus* in Different Maturation Stages.” Carbohydrate Polymers 144: 196–204. 10.1016/j.carbpol.2016.02.051. 27083809

[fsn371275-bib-0033] Li, W. J. , X. F. Tang , X. X. Shuai , et al. 2017. “Mannose Receptor Mediates the Immune Response to *Ganoderma atrum* Polysaccharides in Macrophages.” Journal of Agricultural and Food Chemistry 65, no. 2: 348–357. 10.1021/acs.jafc.6b04888. 27931102

[fsn371275-bib-0034] Liao, B. W. , C. H. Zhou , T. T. Liu , Y. Y. Dai , and H. H. Huang . 2020. “A Novel *Hericium erinaceus* Polysaccharide: Structural Characterization and Prevention of H_2_O_2_‐Induced Oxidative Damage in GES‐1 Cells.” International Journal of Biological Macromolecules 154: 1460–1470. 10.1016/j.ijbiomac.2019.11.027. 31759012

[fsn371275-bib-0035] Lin, J. Y. , Y. P. Chen , T. W. Lin , et al. 2024. “Discovery of a New Compound, Erinacerin W, From the Mycelia of *Hericium erinaceus*, With Immunomodulatory and Neuroprotective Effects.” Molecules 29, no. 4: 1–11. 10.3390/molecules29040812. PMC10891892 38398564

[fsn371275-bib-0036] Liu, C. , S. Gao , B. Liu , et al. 2025. “Study on the CHJ01 Antitumor Activity and Mechanism via Targeting Sphingosine Kinase 1 in A549 Cells.” Pharmaceutical Science Advances 3: 1–9. 10.1016/j.pscia.2025.100077. PMC12709969 41550642

[fsn371275-bib-0037] Liu, J. H. , W. W. Wang , Q. H. Hu , et al. 2022. “Bioactivities and Molecular Mechanisms of Polysaccharides From *Hericium erinaceus* .” Journal of Future Foods 2, no. 2: 103–111. 10.1016/j.jfutfo.2022.03.007.

[fsn371275-bib-0038] Liu, X. P. , Z. Ren , R. H. Yu , et al. 2021. “Structural Characterization of Enzymatic Modification of *Hericium erinaceus* Polysaccharide and Its Immune‐Enhancement Activity.” International Journal of Biological Macromolecules 166: 1396–1408. 10.1016/j.ijbiomac.2020.11.019. 33166554

[fsn371275-bib-0039] Lysakowska, P. , A. Sobota , A. Wirkijowska , and E. Ivanisová . 2025. “Lion's Mane (*Hericium erinaceus* (Bull.) Pers.) as a Functional Component for Wheat Bread Production: Influence on Physicochemical, Antioxidant, and Sensory Properties.” International Agrophysics 39, no. 1: 13–28. 10.31545/intagr/194613.

[fsn371275-bib-0040] Nai, J. J. , C. Zhang , H. L. Shao , et al. 2021. “Extraction, Structure, Pharmacological Activities and Drug Carrier Applications of Angelica Sinensis Polysaccharide.” International Journal of Biological Macromolecules 183: 2337–2353. 10.1016/j.ijbiomac.2021.05.213. 34090852

[fsn371275-bib-0041] Narmuratova, Z. , N. Bisko , K. Mustafin , et al. 2023. “Screening of Medicinal Mushroom Strains With Antimicrobial Activity and Polysaccharides Production.” Turkish Journal of Biochemistry‐Turk Biyokimya Dergisi 48, no. 3: 290–297. 10.1515/tjb-2022-0235.

[fsn371275-bib-0042] Niu, B. , L. Zhang , B. Chen , et al. 2024. “Extraction, Purification, Structural Characteristics, Biological Activities, Modifications, and Applications From *Hericium erinaceus* Polysaccharides: A Review.” International Journal of Biological Macromolecules 291: 1–27. 10.1016/j.ijbiomac.2024.138932. 39706449

[fsn371275-bib-0043] Parada, M. , A. Rodríguez‐Blanco , F. F. D. Magán , and H. Domínguez . 2015. “Sequential Extraction of *Hericium erinaceus* Using Green Solvents.” LWT‐ Food Science and Technology 64, no. 1: 397–404. 10.1016/j.lwt.2015.06.008.

[fsn371275-bib-0044] Park, H. J. , S. Y. Lee , M. Ye , et al. 2023. “Anti‐Obesity Effect of Chitoglucan in High‐Fat‐Induced Obesity Mice.” International Journal of Environmental Research and Public Health 20, no. 1: 1–10. 10.3390/ijerph20010281. PMC9819012 36612600

[fsn371275-bib-0045] Paulauskiene, A. , Z. Taraseviciene , D. Sileikiene , and L. Cesoniene . 2020. “The Quality of Ecologically and Conventionally Grown White and Brown *Agaricus bisporus* Mushrooms.” Sustainability 12, no. 15: 1–10. 10.3390/su12156187.35136666

[fsn371275-bib-0046] Qin, T. , X. P. Liu , Y. Luo , et al. 2020. “Characterization of Polysaccharides Isolated From *Hericium erinaceus* and Their Protective Effects on the DON‐Induced Oxidative Stress.” International Journal of Biological Macromolecules 152: 1265–1273. 10.1016/j.ijbiomac.2019.10.223. 31759000

[fsn371275-bib-0047] Qin, Y. , Z. F. Zhang , T. T. Song , and G. Y. Lv . 2017. “Optimization of Enzyme‐Assisted Extraction of Antitumor Polysaccharides From *Hericium erinaceus* Mycelia.” Food Science and Technology Research 23, no. 1: 31. 10.3136/fstr.23.31.

[fsn371275-bib-0048] Qiu, Y. , G. L. Lin , W. M. Liu , et al. 2024. “Bioactive Compounds in *Hericium erinaceus* and Their Biological Properties: A Review.” Food Science and Human Wellness 13, no. 4: 1825–1844. 10.26599/fshw.2022.9250152.

[fsn371275-bib-0049] Ren, Y. L. , Q. G. Sun , R. N. Gao , et al. 2023. “Low Weight Polysaccharide of *Hericium erinaceus* Ameliorates Colitis via Inhibiting the NLRP3 Inflammasome Activation in Association With Gut Microbiota Modulation.” Nutrients 15, no. 3: 1–17. 10.3390/nu15030739. PMC9920828 36771444

[fsn371275-bib-0050] Ren, Z. Y. , Z. Z. Xu , W. K. Amakye , et al. 2023. “ *Hericium erinaceus* Mycelium‐Derived Polysaccharide Alleviates Ulcerative Colitis and Modulates Gut Microbiota in Cynomolgus Monkeys.” Molecular Nutrition & Food Research 67, no. 3: 1–11. 10.1002/mnfr.202200450. 36443636

[fsn371275-bib-0051] Rui‐Qi, W. , W. Zhi‐Shuo , N. Zhi‐Guo , L. Shan‐Shan , and X. Wei . 2023. “Difference of Active Components Between Fruiting Body and Liquid Fermentation Mycelium of *Hericium erinaceus* and Its Application in Improving Gastric Diseases.” Journal of Food Safety and Quality 14, no. 4: 238–239.

[fsn371275-bib-0052] Shirokikh, I. G. , T. V. Polezhaeva , A. A. Shirokikh , et al. 2020. “Cryoprotective Properties of the Polysaccharide Fraction of the Mushroom *Hericium erinaceus* BP 16.” Biology Bulletin 47, no. 1: 1–6. 10.1134/s1062359020010124.

[fsn371275-bib-0053] Su, Y. , H. X. Li , Z. Y. Hu , et al. 2023. “Research on Degradation of Polysaccharides During *Hericium erinaceus* Fermentation.” LWT‐ Food Science and Technology 173: 1–11. 10.1016/j.lwt.2022.114276.

[fsn371275-bib-0054] Sun, N. X. , Y. N. Zhao , and M. Y. Yin . 2023. “Extraction, Purification, Structure and Bioactivities of Polysaccharides From *Grifola frondosa* (Maitake): A Review.” Journal of Food Measurement and Characterization 17, no. 6: 6200–6213. 10.1007/s11694-023-02133-x.

[fsn371275-bib-0055] Sun, Y. , J. F. Zheng , T. Zhang , et al. 2024. “Review of Polysaccharides From *Citrus medica* L. var. *sarcodactylis* (Fingered Citron): Their Extraction, Purification, Structural Characteristics, Bioactivity and Potential Applications.” International Journal of Biological Macromolecules 282, no. 1: 1–19. 10.1016/j.ijbiomac.2024.136640. 39427793

[fsn371275-bib-0056] Sun, Y. J. , H. Q. He , Q. Wang , X. Y. Yang , S. J. Jiang , and D. B. Wang . 2022. “A Review of Development and Utilization for Edible Fungal Polysaccharides: Extraction, Chemical Characteristics, and Bioactivities.” Polymers 14, no. 20: 1–36. 10.3390/polym14204454. PMC9609814 36298031

[fsn371275-bib-0057] Tan, Y. F. , J. S. Mo , Y. K. Wang , et al. 2024. “The Ethnopharmacology, Phytochemistry and Pharmacology of the Genus *Hericium* .” Journal of Ethnopharmacology 319, no. 3: 1–26. 10.1016/j.jep.2023.117353. 37907145

[fsn371275-bib-0058] Tian, B. M. , Y. Geng , T. R. Xu , et al. 2022. “Digestive Characteristics of *Hericium erinaceus* Polysaccharides and Their Positive Effects on Fecal Microbiota of Male and Female Volunteers During In Vitro Fermentation.” Frontiers in Nutrition 9: 1–17. 10.3389/fnut.2022.858585. PMC9008368 35433782

[fsn371275-bib-0059] Tingting, L. , W. Qin , R. Chuang , et al. 2025. “Targeted Isolation and AI‐Based Analysis of Edible Fungal Polysaccharides: Emphasizing Tumor Immunological Mechanisms and Future Prospects as Mycomedicines.” International Journal of Biological Macromolecules 284, no. 1: 1–15. 10.1016/j.ijbiomac.2024.138089. 39603293

[fsn371275-bib-0060] Tu, J. Q. , H. P. Liu , Y. H. Wen , P. Chen , and Z. T. Liu . 2021. “A Novel Polysaccharide From Hericium Erinaceus: Preparation, Structural Characteristics, Thermal Stabilities, and Antioxidant Activities In Vitro.” Journal of Food Biochemistry 45, no. 9: e13871. 10.1111/jfbc.13871. 34402085

[fsn371275-bib-0061] Wang, J. H. , J. Wu , R. Yamaguchi , et al. 2024. “Uncovering Hericenones From the Fruiting Bodies of *Hericium erinaceus* Through Interdisciplinary Collaboration.” Journal of Natural Products 88, no. 1: 80–85. 10.1021/acs.jnatprod.4c01018. 39723452 PMC11773572

[fsn371275-bib-0062] Wang, X. Y. , M. Wang , J. Y. Yin , et al. 2022. “Gastroprotective Activity of Polysaccharide From the Fruiting Body of *Hericium erinaceus* Against Acetic Acid‐Induced Gastric Ulcer in Rats and Structure of One Bioactive Fraction.” International Journal of Biological Macromolecules 210: 455–464. 10.1016/j.ijbiomac.2022.04.153. 35483513

[fsn371275-bib-0063] Wang, X. Y. , J. Y. Yin , S. P. Nie , and M. Y. Xie . 2018. “Isolation, Purification and Physicochemical Properties of Polysaccharide From Fruiting Body of *Hericium erinaceus* and Its Effect on Colonic Health of Mice.” International Journal of Biological Macromolecules 107: 1310–1319. 10.1016/j.ijbiomac.2017.09.112. 28965966

[fsn371275-bib-0064] Wang, X. Y. , D. D. Zhang , J. Y. Yin , S. P. Nie , and M. Y. Xie . 2019. “Recent Developments in *Hericium erinaceus* Polysaccharides: Extraction, Purification, Structural Characteristics and Biological Activities.” Critical Reviews in Food Science and Nutrition 59: S96–S115. 10.1080/10408398.2018.1521370. 30421988

[fsn371275-bib-0065] Wang, Z. J. , D. H. Luo , and Z. Y. Liang . 2004. “Structure of Polysaccharides From the Fruiting Body of *Hericium erinaceus* Pers.” Carbohydrate Polymers 57, no. 3: 241–247. 10.1016/j.carbpol.2004.04.018.

[fsn371275-bib-0066] Wasser, S. P. 2014. “Medicinal Mushroom Science: Current Perspectives, Advances, Evidences, and Challenges.” Biomedical Journal 37, no. 6: 345–356. 10.4103/2319-4170.138318. 25179726

[fsn371275-bib-0067] Wu, F. F. , and H. H. Huang . 2021. “Surface Morphology and Protective Effect of *Hericium erinaceus* Polysaccharide on Cyclophosphamide‐Induced Immunosuppression in Mice.” Carbohydrate Polymers 251: 1–8. 10.1016/j.carbpol.2020.116930. 33142551

[fsn371275-bib-0068] Wu, F. F. , C. H. Zhou , D. D. Zhou , S. Y. Ou , and H. H. Huang . 2017. “Structural Characterization of a Novel Polysaccharide Fraction From *Hericium erinaceus* and Its Signaling Pathways Involved in Macrophage Immunomodulatory Activity.” Journal of Functional Foods 37: 574–585. 10.1016/j.jff.2017.08.030.

[fsn371275-bib-0069] Wu, F. F. , C. H. Zhou , D. D. Zhou , S. Y. Ou , X. A. Zhang , and H. H. Huang . 2018. “Structure Characterization of a Novel Polysaccharide From *Hericium erinaceus* Fruiting Bodies and Its Immunomodulatory Activities.” Food & Function 9, no. 1: 294–306. 10.1039/c7fo01389b. 29168863

[fsn371275-bib-0070] Xie, B. Y. , L. Yi , Y. T. Zhu , et al. 2021. “Structural Elucidation of a Branch‐On‐Branch β‐Glucan From *Hericium erinaceus* With A HPAEC‐PAD‐MS System.” Carbohydrate Polymers 251: 1–12. 10.1016/j.carbpol.2020.117080. 33142623

[fsn371275-bib-0071] Xie, Y.‐K. , X.‐Y. Pan , X.‐R. Liang , K.‐F. Zhai , and Q. Yu . 2025. “Research Progress on Structural Characterization and Bioactivities of *Poria cocos* and Ganoderma Polysaccharides.” 2, no. 1: 1–15. 10.26599/FMH.2025.9420040.

[fsn371275-bib-0072] Xv, W. , Q. Zheng , Z.‐W. Ye , et al. 2024. “Submerged Culture of Edible and Medicinal Mushroom Mycelia and Their Applications in Food Products: A Review.” International Journal of Medicinal Mushrooms 26, no. 3: 1–13. 10.1615/IntJMedMushrooms.2023052039. 38505899

[fsn371275-bib-0073] Yan, J. K. , Z. C. Ding , X. L. Gao , et al. 2018. “Comparative Study of Physicochemical Properties and Bioactivity of *Hericium erinaceus* Polysaccharides at Different Solvent Extractions.” Carbohydrate Polymers 193: 373–382. 10.1016/j.carbpol.2018.04.019. 29773393

[fsn371275-bib-0074] Yang, Y. , J. H. Li , Q. Hong , X. H. Zhang , Z. M. Liu , and T. H. Zhang . 2022. “Polysaccharides From *Hericium erinaceus* Fruiting Bodies: Structural Characterization, Immunomodulatory Activity and Mechanism.” Nutrients 14, no. 18: 1–16. 10.3390/nu14183721. PMC9503163 36145096

[fsn371275-bib-0075] Yanshree, W. , W. S. Yu , M. L. Fung , C. W. Lee , L. W. Lim , and K. H. Wong . 2022. “The Monkey Head Mushroom and Memory Enhancement in Alzheimer's Disease.” Cells 11, no. 15: 1–17. 10.3390/cells11152284. PMC9331832 35892581

[fsn371275-bib-0076] Yu, P. L. , X. Y. Pan , M. J. Chen , J. S. Ma , B. T. Xu , and Y. Zhao . 2024. “Ultrasound‐Assisted Enzymatic Extraction of Soluble Dietary Fiber From *Hericium erinaceus* and Its In Vitro Effect.” Food Chemistry: X 23: 1–13. 10.1016/j.fochx.2024.101657. PMC11304871 39113740

[fsn371275-bib-0077] Yuki, N. , K. Tetsuyuki , and O. Yukako . 2024. “Comparative Effects of 12 Species of Edible Mushrooms on Colonic Luminal Variables in Rats: Classification of Edible Mushrooms Based on Their Function.” Food Science and Technology Research 30, no. 3: 397–408. 10.3136/fstr.FSTR-D-23-00183.

[fsn371275-bib-0078] Zhang, T. , J. F. Zheng , M. J. Chen , et al. 2024. “A Mini Review of Polysaccharides From *Zanthoxylum bungeanum maxim*: Their Extraction, Purification, Structural Characteristics, Bioactivity and Potential Applications.” International Journal of Biological Macromolecules 282, no. 3: 1–17. 10.1016/j.ijbiomac.2024.137007. 39486707

[fsn371275-bib-0079] Zhu, Y. , Q. Li , G. H. Mao , et al. 2014. “Optimization of Enzyme‐Assisted Extraction and Characterization of Polysaccharides From *Hericium erinaceus* .” Carbohydrate Polymers 101: 606–613. 10.1016/j.carbpol.2013.09.099. 24299817

[fsn371275-bib-0080] Zhuang, H. N. , H. Y. Dong , X. W. Zhang , and T. Feng . 2023. “Antioxidant Activities and Prebiotic Activities of Water‐Soluble, Alkali‐Soluble Polysaccharides Extracted From the Fruiting Bodies of the Fungus *Hericium erinaceus* .” Polymers 15, no. 20: 1–21. 10.3390/polym15204165. PMC10611342 37896408

